# Data Gathering Techniques in WSN: A Cross-Layer View

**DOI:** 10.3390/s22072650

**Published:** 2022-03-30

**Authors:** Omer Gurewitz, Mark Shifrin, Efi Dvir

**Affiliations:** School of Electrical and Computer Engineering, Ben-Gurion University of the Negev, Beer-Sheva 8410501, Israel; markshi@post.bgu.ac.il (M.S.); efid@post.bgu.ac.il (E.D.)

**Keywords:** wireless sensor networks (WSNs), Internet of things (IoT), data gathering, wearables, compressed sensing, network coding, mobile sink, energy harvesting

## Abstract

Wireless sensor networks (WSNs) have taken a giant leap in scale, expanding their applicability to a large variety of technological domains and applications, ranging from the Internet of things (IoT) for smart cities and smart homes to wearable technology healthcare applications, underwater, agricultural and environmental monitoring and many more. This expansion is rapidly growing every passing day in terms of the variety, heterogeneity and the number of devices which such applications support. Data collection is commonly the core application in WSN and IoT networks, which are typically composed of a large variety of devices, some constrained by their resources (e.g., processing, storage, energy) and some by highly diverse demands. Many challenges span all the conceptual communication layers, from the Physical to the Applicational. Many novel solutions devised in the past do not scale well with the exponential growth in the population of the devices and need to be adapted, revised, or new innovative solutions are required to comply with this massive growth. Furthermore, recent technological advances present new opportunities which can be leveraged in this context. This paper provides a cross-layer perspective and review of data gathering in WSN and IoT networks. We provide some background and essential milestones that have laid the foundation of many subsequent solutions suggested over the years. We mainly concentrate on recent state-of-the-art research, which facilitates the scalable, energy-efficient, cost-effective, and human-friendly functionality of WSNs and the novel applications in the years to come.

## 1. Introduction

Wireless sensor networks (WSN) are data measurement and gathering networks based on small hardware (HW) units capable of sensing, monitoring, or measuring their surroundings. The sensed data are transmitted directly or by relay via other sensors to some sink or server or a base station. The ultimate objective of such a configuration is to provide control or exploration capabilities over an area where the network is deployed. WSN characteristics can vary substantially: they can be composed of a few to hundreds of thousands of sensors; the monitored terrain can range from a small coverage area (e.g., the human body) to a vast realm (e.g., a forest area for fire detection); the sensed variables of interest of the surroundings are diverse (e.g., weather or health parameters, acceleration, pollution); and the sensors can have different characteristics (e.g., size, computational power, energy source).

The Internet of things (IoT) aims to improve day-to-day life. The concept includes smart cities, smart homes, pervasive health care, assisted living, environmental monitoring, surveillance, and so on. The IoT paradigm relies on interconnecting a large number of devices (things) linked by the Internet via heterogeneous access networks through which they can exchange information with one or more Internet gateways that can process the data, take action, and forward them to another destination if needed. Since many IoT devices are expected to be wireless, and since sensing is one of the main tasks and tools utilized by the IoT paradigm, IoT systems will rely extensively on WSN technology. The scale of scenarios where WSN are deployed nowadays is vast. Traditionally, WSN were classified based on their placement (e.g., terrestrial, underground, multimedia) [1]. Since WSNs are closely associated with IoT, contemporary classification tends to re-attribute the notions of the WSN domain to the IoT domain [2] and classify them based on their primary objectives, such as smart cities [3,4], healthcare [5], retail and leisure [6], utilities (e.g., smart home energy control, water metering and leak detection, and other general infrastructure monitoring networks) [7], agriculture and environmental safety (e.g., smart farming and harvesting, pest control [8,9,10], seismology monitoring [11,12], oceanology [13]), and more.

As previously explained, one of the main tasks of both WSN and IoT systems is data collection and dissemination. Reports are collected from the devices, and updates and operational assignments are distributed. Maintenance and functional assessments are also collected and disseminated. Data collection and dissemination in very dense networks such as WSNs and IoT networks which span heterogeneous devices, a significant percentage of which are expected to be small, with very constrained processing, storage, and energy resources and with minimal network capabilities, is challenging and draws significant attention both by the industrial and academic communities. Some of these challenges include: (i) Information management — the amount of information collected or needing to be disseminated to the relevant entities is enormous, and some is expected to be redundant, both in terms of the information sent by each device, which can be compressed, and in terms of same information received by different entities. Accordingly, innovative techniques are required for data compression to reduce transmitted data over wireless channels and aggregation techniques that exploit the redundancy between information sent by the different entities. (ii) Data analysis and reaction — the expected vast data exchange and the low latency requirement (at least for some of the information collected) require processing and analysis of data in real-time or near real-time, to enable timely decision making and instantaneous action-taking.

The ability to successfully transmit and gather vast streams of data incoming from an enormous number of devices and sensors and finally to successfully analyze them, in order to automatically control a much larger scope of everyday life systems, directly couples the process of data gathering with Big Data related challenges (e.g., [14,15,16,17]). Furthermore, leveraging Cloud Computing platforms offers significant advances in data analytical abilities (e.g., [18,19,20]). It provides new horizons to further develop and increase the size of WSN/IoT networks both in the sense of the number of sensing units and in the sense of the amount of the acquired data (e.g., [21,22,23]). (iii) Connectivity — collecting and disseminating data from and to many devices, potentially through vast, dense, heterogeneous networks, will be one of the biggest challenges of the future of IoT; accordingly, novel MAC protocols and coding schemes should be devised to comply with this challenge. With this respect, air time utilization and energy efficiency are of primary importance for the MAC layer protocol design. Any MAC layer protocol should ensure that devices utilize the wireless channel frugally and with minimum energy consumption. (iv) Security and Privacy — Connecting enormous numbers of devices to the Internet exposes the IoT network to serious security vulnerabilities. All the more so since the relevant entities are limited. Accordingly, issues such as authenticity, data encryption, and vulnerability to attacks (e.g., device impersonation) are critical for the IoT paradigm’s continuous growth (e.g., [24]). In addition, since the information transmitted over the WSN and IoT networks can be highly confidential (e.g., health reports, device tracking), the collection and dissemination of this information create significant challenges related to data protection and privacy.

This survey will explore the state-of-the-art of data collection and dissemination aspects in WSNs and IoT environments mentioned above. We will review essential milestones yet mainly focus on recent publications and present the new trends and research directions. Our resources included mainly Google Scholar, IEEE Xplore and our university’s library databases, utilizing the keywords of this paper. We also used important references from the bilbiography of the initial papers and ones that cited them. Data collection spans all the networking layers, from the physical implementation of transmitting bits across a communication medium to the application layer. Due to the wide-ranging scope of the topic, we will not be able to cover all its aspects (for example, in this paper, we will not discuss the critical topic of security and privacy). Some of the issues will be covered more thoroughly than others. However, since some of the topics we discussed rely on the general wireless communication technology and on broad setup protocols which are not data-gathering oriented per se, on some of the topics, we will provide a more comprehensive background and describe protocols that are aimed at a broader domain than data-gathering. For example, many medium access control (MAC) and wireless routing protocols are designed for a wide range of topologies, traffic patterns, quality of service requirements, etc. Even though they can be applied, they are not explicitly designed for data gathering. We will include some more general yet essential studies in our survey. To grasp the whole picture and to better understand some data-gathering-related issues, in some cases, we will delve into the pertinent background and stray into some peripheral topics. We will cover topics related to all layers of the protocol stack. Sometimes classification based on a stack is not clear-cut, as some of the issues involve multiple stacks.

In particular, the survey comprises the following topics:

The device’s platform, which accommodates the sensing unit, can highly impact the performance of the application utilizing it and specifically the data-gathering application, and vice versa, the application (e.g., data gathering) can impact the platform architecture when designed in application-oriented manner or when some of the essential features and requirements are taken into account in the platform design process. The same mutual effect also applies to the WSN infrastructure and the network architecture (e.g., topology, system organization). We start with reviewing studies pertaining to the general device’s platform and infrastructure-related novelties (Section 2). We cover new domains which were only recently exposed to WSN and IoT networks and introduced new opportunities for algorithm design in such networks. Some of these novel technologies have revolutionized the way applications can utilize each particular device as well as the shared network and have enabled new algorithm opportunities and design challenges across the entire protocol stack which we describe throughout this survey.

Next follows Section 3 which provides a focused summary of recent advances in compressed sensing—a signal processing technique that can take advantage of sparsity and redundancy of the data. In the context of data gathering procedures, compressed sensing is utilized to reduce report payload at several levels, which include reduction of the sensed data size and the transmitted report size, by pruning the devices that need to send reports and by compressing the combined relayed data before forwarding it toward its destination (the sink). We provide a basic compressed sensing background and review the state-of-the-art in the context of data gathering in WSN.

Section 4 considers the medium access control (MAC) sublayer. In wireless communication, channel utilization is critical and broadly influences several performance aspects such as throughput, latency, power utilization, delivery ratio, and more. Over the years, numerous algorithms and protocols have been suggested for wireless channel access in general, and WSNs with their specific characterization in particular. In Section 4, we review only a small fraction of the countless MAC protocols that have been designed for WSNs. We mainly concentrate on protocols that highlight a conceptual approach or trend and review some of the more recent MAC protocols in data gathering in WSN and IoT networks, which address new challenges such as highly dense networks, congested channels, and very limited resources.

We ascend the protocol stack and in Section 5 we address routing aspects. As with the MAC sublayer, routing protocols in multi-hop wireless networks have also been extensively studied. We start by providing several milestones in the context of data gathering in WSNs. We continue with more recent studies which mainly include enhancements to the aforementioned protocol, taking into account new challenges such as scalability demands and energy-related advances which present new opportunities yet impose new constraints. We continue with studies that leverage the multi-hop topology to realize a network-coding mechanism. Finally, we discuss a new paradigm that extends the traditional setup in which the sensed data need to be routed toward a static central monitoring station (sink), and utilize a mobile sink (or sinks) that traverse the terrain and can help in collecting the devices’ reports. We review several state-of-the-art schemes in this mobile sink paradigm.

The final section of this survey is dedicated to wearable technology in the form of smart devices that are attached to the human body to monitor the user and their environment. Wearable technology involves challenges in all the aspects discussed in the previous sections, yet they introduce new opportunities for high-demand applications with unique performance requirements and constraints. Even though we do not attempt to provide a comprehensive review of the numerous applications suggested over the years, we emphasize this prominent application layer and discuss several applications in Section 6.

**Figure 1:**
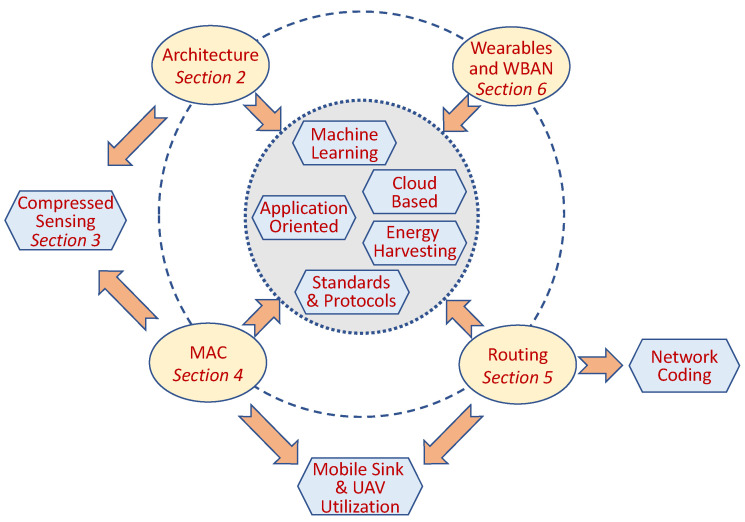
Research directions. While we organize the sections according to the layers, this diagram shows how research directions are connected across different layers. The ovals denote the major research areas (which are associated with sections in the paper), and the hexagons refer to more specific sub-areas, technological innovations, and research tools. The arrows represent a schematic inter-relation between them.

As previously explained, we roughly partitioned the topics covered in this survey based on the communication layers and organized the sections accordingly. We note that this partition is somehow artificial, as many innovations in data-gathering involve more than one layer. Furthermore, many technological advances and research areas affect multiple domains in different layers and are visited in more than one section. Figure 1 depicts the schematic structure of the paper. In the figure, the ovals represent the main research domains covered in the paper. The hexagons represent the most prominent research tool innovations and techniques utilized by data-gathering, which are covered in the paper. The arrows represent the inter-relation between them. For example, technologies such as energy harvesting (EH) or Machine Learning (ML) and Artificial Intelligence (AI) are utilized by innovations in all the layers starting from the platform hardware to the application layer. However, network coding is mainly utilized by the network layer. Unmanned Aerial Vehicle (UAV) is leveraged by both the MAC and the Network layers.

## 2. WSN Architecture—Arising Platforms and Novel Infrastructure Concepts

Our primary focus in this survey is data gathering in the context of wireless communication networks. The units that generate the data (typically sensors) are application-dependent and can serve a large variety of realms, e.g., health, environmental, activity monitoring, etc. Even though the sensing unit is the essence, we will not cover it thoroughly, and we will only skim through it sporadically when discussing applications and their specific requirements. Nonetheless, the term “sensor” typically refers to the whole platform or device in which the sensing unit is only one component out of many, such as processing unit, transceiver unit, power unit, antenna, and more, several of which can be integrated into the device according to the particular application needs. The sensing unit itself has its own requirements and constraints, and in many situations cannot be altered. In addition, the integrated unit architecture and the platform design can be subject to various stringent constraints. For example, size requirements can impose a strict constraint on the device design; low power consumption, low production cost, and self-operation can represent additional constraints. Accordingly, the device architecture is fundamental and affects many other factors in the system. For example, power supply affects the life span (or the time needed to replace the batteries); it also affects transmission range, memory, and processing unit, which in turn can affect the algorithms that can be executed on the device, etc.

**Figure 2:**
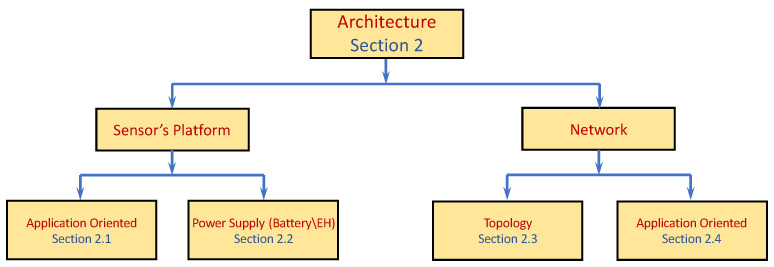
Schematic description highlighting the main topics covered in the section.

Extensive research has been conducted on the design and architecture of the end device and the infrastructure. We leave a detailed description of the basic components such as the sensing unit, transceiver, antenna, processing unit, etc., as well as the underlying hardware beyond the scope of this survey. To this end, the objective of the rest of this section refers to how data-gathering objectives may impact both the design of specific sensors and the WSN infrastructure. By the latter, we mean topology, system organization used to gather the data, and algorithms to implement the data gathering. It is noteworthy that the sensors’ characteristics also dictate the topology and, consequently, the data aggregation algorithms. In the sequel, we mention several platform architecture designs as well as several network-wide architectures, mostly from recent years. Additional similar studies appear throughout the survey, yet they are organized in chapters according to the area in which they propose the most significant novelty. Figure 2 presents a schematic description of the section. Since several papers presented in this section cover more than one topic, and since, as previously mentioned, this section is not presumed to provide an exhaustive list of all papers or topics covered by the scope of WSN architecture, and some of the topics are not covered at all or covered by only a few representative papers, the description is broad and only highlights the main topics covered in the section.

### 2.1. Application-Oriented

Many sensor platforms are application-oriented. Occasionally, their suggested architecture can be applied to other applications; however, their design and evaluation are typically aimed at a specific one. Hence, in many cases, both hardware and software technological developments are introduced for effective functioning. One of the most common tasks of WSN is the obvious one of monitoring a terrain. There are many variants of WSN monitoring. For example, the requirement can be to monitor every point in the Field of Interest (FoI) vs. monitoring a limited number of specific locations or targets (aka target coverage) vs. just monitoring a border of a region to detect intruders (aka barrier coverage). The coverage problem typically involves selecting a subset of sensors that fulfill the monitoring objective while maintaining network connectivity. The sensors’ capabilities and the monitoring objective determine the network topology.

We present several recent examples that mainly concentrate on connectivity and data gathering under the constraints of the monitoring objective. Biswas et al. [25] focus on energy-efficient data gathering in target coverage problem, in which an *n* sensor WSN needs to monitor *T* specific targets, and there exists a route (multi-hop) from each source to the sink. The paper assumes that the source nodes that sense the targets and initiate data packets into the network are known, and deals with the forwarding of these packets to the sink. The paper proposes a distributed data gathering algorithm in which after each node discovers its neighbors and their hop-count to the sink, it will forward data packets (when required) to its neighbor with maximum remaining energy and a lower hop count to the sink (the remaining energy is assumed to be known). Ammari [26] focuses on the *k-coverage* problem in which each point in the FoI is required to be covered by at least *k* sensors at any time, and each active sensor participating in the monitoring task is required to be connected to the sink (possibly via a multi-hop route). The paper assumes that the sensors are heterogeneous (they do not have the same characteristics) and mobile, hence the sensors can move toward any region of interest in the deployment field to participate in any deficient *k-coverage* area and can also act as mobile proxy sinks that collect sensed data from the sensors and deliver them to the sink. Ammari [26] partitions the problem into two problems which are solved sequentially. Namely, the *mobile k-coverage* problem, which selects a minimum subset of active sensors that solve the *k-coverage* problem and the *data gathering* problem, and devise a forwarding scheme from the active sensors to the sink such that the energy consumption due to sensor mobility and communication is minimized.

Mdemaya and Bomgni [27] utilize mobile sensors to achieve area coverage. These mobile sensors can be moved and relocated to cover holes after the random deployment. The authors suggest a two-phase approach. According to the first one, the monitoring area after the initial random deployment is identified (by the BS), and mobile nodes are relocated to cover the monitoring holes detected after the initial deployment, trying to ensure full coverage of the AoI by the static and relocated sensors. At the second stage, the proposed algorithm schedules the sensors’ activity (awakening and transmission times) that minimizes the energy consumption of the nodes while collecting and sending data to the base station. To this end, the paper distinguishes between “normal” nodes and cluster heads. A survey that reviews algorithms and techniques related to the connectivity-coverage issues in WSN can be found in Boukerche and Sun [28].

Occasionally, WSN architectures and designs are more application-oriented. For example, Cerchecci et al. [29] propose a sensor node topology that uses low-cost and low-power components for energy-efficient waste management in the context of smart cities. The architecture described in [29] suggests a node architecture for measuring the filling level of trash bins and utilizes LoRa LPWAN (low-power wide-area network) technology for real-time data transmission to collect the measured data in a remote data collection center. The design of a sensor node that can detect the presence of water on home floors and provide early warning of water leaks is suggested by Teixidó et al. [30]. The paper presents and deploys both hardware and software of the network components (flood sensing nodes, actuator nodes, and a control central); communication within the sensor network relies on the IEEE 802.15.4 standard. Borrero and Zabalo [31] present a low-cost agriculture-oriented system. The suggested system is based on LoRa technology and can collect various measurements, such as humidity, ambient temperature, soil moisture, and temperature, and enables a farmer to access all of the information necessary to achieve efficient irrigation management of crops in real time. The developed wireless sensor node has been optimized both in hardware and software and exhibits very low power consumption.

### 2.2. Energy-Harvesting (EH)

One of the main concerns of the sensor platform’s design is the source of energy. Typically, the energy source is a battery attached to the sensor platform. It is utilized to provide power to all the required operations, e.g., wireless transmission, computation, memory, etc. The battery properties (e.g., technology used and size) can determine its lifespan as well as several other properties, e.g., transmission range. In many systems, the battery is a burden, as it increases the cost of the system, constrains the platform size, and most importantly, requires to be replaced occasionally. The challenge of saving power spans all the protocol stack; energy considerations show up in each part of this survey. As with the other layers, PHY layer innovations have also been suggested as to how to utilize battery power efficiently.

An alternative approach to overcome the battery hurdle is to embed a mechanism that harvests energy. Such a mechanism can be embedded alongside the battery to extend its lifespan, or more commonly, it can completely replace the battery so that all the functions rely on it. Batteryless WSNs that rely solely on energy-harvesting (EH)-WSN can compromise performance; for example, their transmission range can be shorter, the available energy can constrain their awake time, and so on. One of the main challenges is to locate the ambient resource from which the energy can be harvested. Many studies have explored different energy sources that can supplement energy, such as solar, vibration, wind, motion, electromagnetic, and more. Numerous comprehensive technological overviews with their advantages and limitations, energy harvesting modeling, challenge expectations, and prospects can be found in, for example, Refs. [32,33,34,35,36,37]. A more recent system design review on battery-free and energy-aware WSNs, which utilize ambient energy or wireless energy transmission, is given in [38]. It addresses energy supply strategies and provides insight into energy management methods and possibilities for energy saving at the node and network levels.

Khalid et al. [39] suggest a zero-power wireless sensor architecture that consists of a capacitive sensor (a sensor that associates the parameter of interest with the change in the capacitance), an RFID chip, a circulator (allows power flow between three defined ports), and an antenna (batteryless). The conceptual idea is that the sensor reflects the signal received from the RFID, with a change in phase, which is relative to the sensed value. Design and implementation of an energetically autonomous WSN platform for ambient monitoring in indoor environments are suggested by Abella et al. [40]. The proposed self-powered autonomous sensor node platform relies on embedded photo-voltaic (PV) panels to harvest the energy, a microcontroller and an RF transceiver with an attached antenna. The suggested architecture was prototyped and validated experimentally. Lee et al. [41] propose a floating wireless device with energy harvesting capability. The floating device is energetically self-sustaining for extended operational hours. It supports long-range communication between wireless sensor nodes and a gateway relying on the LoRa technology while deployed over a water surface. The floating device can be used as an environmental monitoring station to remotely collect weather and water quality information. Ref. [42] present the design of a wireless sensor node, powered by solar energy, that collects environmental data and can transmit it across vast distances (directly to the cloud). The architecture presented therein relies on low-power wide-area network (LPWAN) protocols that provide a long-range communication system with limited data to transmit and high energy efficiency. The authors utilize Sigfox technology in their proof-of-concept design.

As previously mentioned in numerous papers, surveys and tutorials exploring different aspects of energy harvesting in WSN exist (a sliver of which we present herein). We will revisit EH when we discuss various aspects of data aggregation, such as routing enhancement for EH-WSN (under EH constraints), on which we elaborate in Section 5.2 or when discussing wearables in Section 6.

### 2.3. Topology

Throughout the survey, the interaction of WSN and IoT will arise in multiple contexts. While this survey mainly deals with data gathering by means of wireless units, an IoT unit presumes a more high-level entity for localized data gathering. To assess the connection between these two concepts, the reader is advised to refer to the most recent work by Devadas et al. [43], for example, where the authors enumerate the IoT data management frameworks, challenges and issues. The chapter focuses on three layers of data management in IoT networks, communication, storage and processing. In addition, deployment of IoT Data management for *smart home and smart city* is described.

It is essential to distinguish between a *one-directional* WSN platform, where sensors merely gather the data and activate a specific infrastructure and set of technologies to further send it to a sink, and a *bi-directional* WSN platform, where the sensors are expected to be able to act according to control messages received from a sink. In the latter case, the sink might be a higher-level entity (e.g., a cloud-based server). While the general data-gathering techniques are usually agnostic of the control direction, additional constraints might be imposed. Delay of the responses, latency, BW usage efficiency, security, and privacy are some of the demands to consider. Another example of a bi-directional platform can be seen in social sensor clouds (SSC), which connect a social network with a sensor network via a cloud infrastructure. See, for example, Zhu et al. [44], which presents a scenario of a smart village and provides discussion on various aspects including green planning, energy concerns, and speed of data gathering and sharing. In Dinh and Kim [45], an on-demand WSN platform is designed. The authors suggest a data-gathering protocol that addresses bandwidth consumption and delivery latency and minimizes the number of requests to save resources. An infrastructure where sensors form groups belonging to private owners constitutes a special case. This may be the case in a smart city environment; this means that privacy and/or security considerations should be prioritized. This is the topic addressed by Zhu et al. [46]. The authors provide a trust-assisted cloud for WSN but have throughput issues in mind. Kuo et al. [47] suggest a WSN-based IoT platform that provides a reliable connection between sensors in the field and the database on the Internet. The proposed platform is based on the IEEE 802.15.4e time-slotted channel-hopping protocol with resource-constrained devices supporting heterogeneous applications. The paper suggests a scheme that compensates the clock drift for every timeslot to maintain the clock synchronization required for the time-slotted channel-hopping protocol.

Edge computing, as discussed by Satyanarayanan [48], allows distributing the data gathering burden across multiple cloudlets, which might be highly beneficial for large WSN. This platform paradigm aims to improve many important aspects: reduced latency of data delivery, increased bandwidth, scalability, resilience to possible cloud outages, and privacy control. However, the platform presumes an initial capital investment and later maintenance.

A virtual sensor network was proposed by Abdelwahab et al. [49]. Once a user-initiated sensing request is dispatched to a cloud, a suitable set of sensors is found for the task. The decision is made according to the cost function, which depends on the specific (e.g., monetary) cost of using sensors from the designated set, the benefit that can be received from using these sensors, and their effectiveness in distances and delays (calculated, e.g., in number of hops from sensor to a sink/gateway), also expressed as virtual links. The cost might be customized, while a general virtualization problem is formulated and the algorithm is provided.

Integration of unmanned aerial vehicles (UAVs) and WSN for crop monitoring in precision agriculture is described by Popescu et al. [50]. The authors suggest a down-up scheme, where the collected data is hierarchically processed from the ground level to the cluster head (CH) level, then collected by the UAV level and finally delivered to the cloud for analysis and possible feedback. Particular emphasis is put on outlying measurements from specific sensors, as they can indicate either a possible sensor failure or an upcoming unusual event inside the agricultural field. The measured data were processed through a consensus algorithm. Concurrently, it suppressed outlier values left for further examination for the cloud-based analysis. In addition, this study focused on the UAV trajectory planning to collect the data observed by the WSN. Actual deployment with several tens of sensors and several CHs is provided and analyzed. Note that we dedicate Section 5.4 to data gathering assisted by a mobile unit.

An implementation of a ubiquitous *consumer data service* for transmitting short messages to any computing platform is provided by Datta et al. [51]. The authors demonstrate a data cycle model that allows any device with sensor(s) to report data encoded in short messages. The raw data reaches a central or distributed computing platform, where it undergoes transformation and evolves into rich and structured valuable information for higher-layer applications. The proposed data cycle model and DataTweet architecture are aimed at smart city and large-scale crowd-sensing-based IoT scenarios.

### 2.4. Application-Oriented Network Architecture

We continue by covering special types of WSN platforms for data gathering and specialized application-driven architecture types. Ayele et al. [52] suggest an IoT network architecture for *wildlife monitoring systems* (WMS) for scenarios in which animals exhibit sparse mobility, which results in sporadic wireless links. In addition, they suggest a data forwarding enhancement that adopts the flood-store-carry-and-forward paradigm suggested in the seminal ZebraNet study by Juang et al. [53], in which in order to send data to the sink, the nodes disseminate it among themselves until it reaches the sink. Specifically, each node stores the data needing to be conveyed, waits for connectivity with other nodes, and distributes the data to them, and they repeat the same process. Accordingly, the data is spread throughout the entire network (i.e., flooding) and will eventually be received by the sink. The authors in [52] suggest leveraging locally available routing parameters to improve opportunistic data forwarding algorithms by managing the data replication decision.

Saleh et al. [54] suggest extending the lifetime of a wireless sensor network used in mobile healthcare applications by increasing the number of bits transmitted per symbol, and specifically to rely on a quaternary interconnect scheme in which each transmitted symbol modulates two bits. A complementary neural network, static RAM-based architecture is suggested to reduce energy consumption in storage and transmissions during the data dissemination process. A WSN dedicated to home deployment for elderly healthcare and early health emergency alarm is discussed by Alsina-Pagès et al. [55]. The authors first raise privacy concerns related to the monitoring, and accordingly, advocate that only sound-based surveillance aimed to merely indicate alarming situations is appropriate. In order to further conform to the privacy demands, they focus on distributed architecture (rather than on a centralized one), where each of the WSN sensors sends encrypted identifiers of their measurement. The identification of events is built on feature extraction. This is done on the frequency domain by first dividing the incoming signal into blocks with Hamming sliding window, then transforming into the frequency domain using Discrete Fourier Transform (DFT) to evaluate the contribution of every band of the spectrum. The final coefficients are obtained after Discrete Cosine Transform (DCT). The conclusive parts of the proposed algorithm classify the coefficients, feeding them into Support Vector Machines which classifies the estimated audio event. The authors assert that the classification results could be further improved by incorporating a deep artificial neural network (ANN) into their system.

In AbeBer et al. [56], a similar method was implemented for urban noise monitoring. Namely, while STFT was utilized for the noise preprocessing, the classification of noise levels and events was performed by convolutional neural networks (CNNs). The authors used several previously published networks; see references therein. Similar methods for noise monitoring WSN were introduced by Siamwala et al. [57]. The frequency-domain analysis was performed. Then, classification by statistical methods was accomplished (Gaussian mixture model was used). In addition, the authors in [57] provide an elaborate WSN architecture, where energy-harvesting solar panels augment the sensors’ lifetime and the sensors’ state-of-charge is transmitted and tracked by central, more powerful nodes.

## 3. Compressed Sensing

Many data-gathering applications rely on numerous self-powered smart devices to collect real-time data and convey it via a wireless medium to a central entity or entities (e.g., the cloud) to further process it and act upon it. Such devices are expected to perform two basic operations—sensing and wireless connectivity. Two important hindering aspects that derive from these operations influence the performance and need to be considered are: (i) energy consumption associated with these two operations, especially when many of these devices are typically simple with limited computation abilities and battery lifetime (ii) airtime utilization, which can also degrade the performance causing high delays, jitter, battery consumption, etc. Accordingly, one of the main challenges in combating these limitations is reducing the report payload, which affects each report’s transmission time and channel utilization. Reducing the payload of the sensed data can be accomplished at different levels; it can be done in the sensing stage by reducing the size of the sensed data, as well as in the report preparing stage by compressing the report size, and in the transmission stage, by selecting which devices need to send reports, thus limiting redundant information. When the reports need to travel multiple hops before reaching their destination, this can be done at the relay stage by pruning, unifying, and compressing reports. In the sequel, we discuss Compressed Sensing (CS). This novel paradigm can reduce report payload at several levels mentioned above, hence lowering the sensing operation’s transmission time and energy consumption.

Compressed sensing is a signal-processing technique that is most advantageous when the subject signal is sparse in some domain, such that a minimal non-zero vector of coefficients can represent it. The signal sparsity enables a high-quality reconstruction, which is attained by finding the solution to an under-determined linear system of equations with the smallest possible number of non-zero values. Thus, a convex minimization problem needs to be solved to perform the recovery. Note that the CS technique performs non-uniform sampling of the data signal with an average sample rate usually smaller than the minimal rate mandated by the Nyquist–Shannon sampling theorem. A detailed view of the technique can be found in Balouchestani et al. [58] and Donoho [59]. Various networking domains can utilize compressed sensing, for example, Feizi et al. [60] depict some applications of CS over networks and elucidate the connection between CS and traditional information-theoretic techniques in source coding and channel coding. Particularly, CS is highly suitable for sensed data gathering in wireless sensor networks (e.g., physical phenomena or a scenery), as it can leverage the expected high spatial and temporal correlation between sensing reports sent by neighboring sensors at different times in order to acquire the CS paradigm’s desired sparsity. In the following, we review several such data-gathering schemes that utilize CS.

Luo et al. [61,62] consider a densely deployed monitoring sensor network in which reports traverse multiple hops before reaching their destination, a sink. These studies rely on the concept that sensors’ readings are spatially correlated; hence, there exists a transform domain in which the sensed signals can be sparsely represented. Both propose a compressive data-gathering (CDG) procedure in which sensors distributively encode their reports by projecting them on a random space basis using random coefficients. These encoded reports can be decoded at the sink using compressive sensing techniques. Specifically, CDG is designed for multi-hop networks where messages need to travel multiple hops before reaching their destination. The sampling process that characterizes the CS compression process is performed individually at each sensor by simple multiplications and additions. Particularly, CDG suggests that rather than forwarding individual sensor readings, each sensor uses each of its reports (measurements) to construct and send *M* different messages, each comprising a weighted sum of the sensor’s own report with other sensors’ reports traversing it (relaying). Formally, denote by vector d$\;={\left[d_{1},d_{2},\ldots ,d_{N}\right]}^{T}$ the measurements (readings) obtained by all the sensors, where *N* is the number of nodes in the WSN and $d_{i}$ denotes the measurement (reading) obtained by sensor $s_{i}$. The sink obtains *M* messages (weighted sums) represented by the vector $y=\Phi d=\phi_{1}d_{1},\phi_{2}d_{2},\ldots ,\phi_{N}d_{N}$, where Φ is an M×N(M<<N) matrix comprising the series of coefficients generated by the sensors, and in particular, $\phi_{i}$ is the *i*-th column vector of Φ, which denotes the random coefficients selected by sensor *i*. Luo et al. [61] suggest that the measurement matrix (coefficients matrix) Φ should be a full random matrix with its entries being *i.i.d.* Gaussian random numbers drawn according to $N(0,\frac{1}{M})$. The paper suggests that each weighted sum coefficient be chosen pseudo-randomly based on each sensor’s ID in order to avoid the burden and high overhead required to collect these coefficients by the sink if they are chosen randomly. Ref. [62] extends the random coefficient selection suggested in [61] to an only partly random matrix in which the entries of a sub-matrix are still drawn according to $N(0,\frac{1}{M})$. Yet, for the rest of the matrix, two options are suggested, either an upper triangular matrix with non-zero entries drawn according to $N(0,\frac{1}{M})$, or the identity matrix. CDG exploits the compressive sampling theory and shows that when the sensor readings are compressible, the sink will be able to accurately recover the reports even when the number of weighted sums (messages) each sensor generates for each report (*M*) is much lower than the number of reporting sensors (*N*). For example, on a route comprising *N* sensors, the sink needs to collect only M<<N messages to encode the information sent by all the *N* sensors.

Several studies further explore the sparsity of the sensed signal and its projection matrix, as well as the number of messages (*M*) that should be delivered to the sink. For example, Wang et al. [63] argue that most natural signals are nonstationary and ordinarily variable in the temporal and spatial domains. In CS, these directly influence the reconstruction process and the number of required measurements; consequently, setting a fixed number of measurements with a fixed transform basis (coefficient matrix Φ) can result in poor performance (inaccurate measurement reconstruction). Accordingly, Ref. [63] suggests an adaptive data-gathering scheme based on CS, which utilizes an autoregressive (AR) model to exploit the local spatial correlation between sensed data of neighboring sensor nodes. The suggested reconstruction scheme adapts to the variation of sensed data by adjusting the AR parameters. The number of measurements is adjusted adaptively with the sensed data by evaluating the recovery result and approximating the number of measurements required to satisfy the accuracy demand.

To reduce the transmission overhead, Xu et al. [64] propose the compressed sparse function (CSF) scheme. The basic concept of CSF is, rather than encode the sensed data by projecting it on a basis on which it can be represented sparsely, as in typical CS-based schemes, to compress the sensed data in the form of sparse functions, which are sent to the source. The source can recover the function using techniques from polynomial approximation/interpolation theory and use them to compute data values that were not reported. Specifically, CSF finds a function that maps the sensors’ identifiers and their readings, which can be expressed in a very sparse way, and only communicates this function to the sink. After the sink recovers the function, it can recover all the *N* sensor readings. Xu et al. [64] show that the CSF approach can provide good recovery accuracy (better than the CDG scheme suggested by [61]) while substantially reducing the message overhead (mainly in tree-structured networks). Li et al. [65] present a general CS framework for WSNs and IoT and show how the proposed framework can be utilized to reconstruct the compressible information. The suggested framework comprises three phases: (i) information sensing to detect and compressively sample event signals; (ii) compressed sampling, in which the system samples information traversing the networks; and (iii) reconstruction algorithms, in which the system accurately reconstructs the original signal from the compressed samples.

Different studies tackle the sampling issue and suggest different approaches to reduce the number of reports sent such that only a subset of the sensors sense the object or phenomenon at a time. Several studies explore how sensed data is conveyed to the sink with the insight that the compression is at least attained along the path to the sink, and is therefore affected by it. For example, Dhanapala et al. [66] show that a random-walk-based sampling, rather than the conventional uniform-sampling-based CS for function recovery, can be used for phenomena awareness either at a sink or at other sensors without a sink, with minimal additional sampling. As the distribution of the samples has a significant effect on the recovery, Ref. [66] suggests an upper bound for the probability of successful recovery with a given error percentage. The derived bound provides an approximate number of samples required to recover a function under a selected basis and a sampling scheme. Zheng et al. [67] further argue that random walk provides a more practical approach for the data-gathering application in WSNs and explores the sparsity of collecting non-uniform measurements while sampling along multiple random paths. The paper suggests that the M×N measurement matrix will be characterized by *M* independent random walks. Specifically, each of the *M* matrix rows corresponds to the set of vertices visited by the respective random walk. The paper analyzes the required number of random walks (*M*) and their corresponding lengths (how many entries on each row are non-zero) under the proposed random walk algorithm.

Zheng et al. [68] suggest a cluster-based data-gathering mechanism, in which the terrain is divided into cells; in each cell, a node is randomly selected as the cell head, which collects the data from the cell members and forwards it to the sink. Zheng et al. [68] suggest two forwarding mechanisms, one relying on centrally defined tree-based forwarding, and another that is a gossip-based approach. The projection process is similar to Luo et al. [61,62], being based on random coefficients. Another clustering-based hierarchical data aggregation protocol that relies on CS, termed HDACS, is suggested by Xu et al. [69]. Specifically, HDACS constructs a multilevel hierarchical structure and adaptively sets multiple compression thresholds based on cluster sizes at different levels of the data aggregation tree to optimize the amount of transmitted data. The encoding procedure is adapted from [62], where each cluster-head recovers (decodes) all received messages from its descendants (retrieves the original data) before compressing and sending it to its parent cluster-head.

Motivated by reducing power consumption, Lan and Wei [70] also suggest a compressibility-based clustering algorithm for hierarchical compressive data gathering. In this study, the network is decomposed into a logical chain, and sensor nodes are grouped based on the compressibility of their readings instead of by a random clustering approach. This clustering approach strives to minimize the average compression ratio of all clusters by greedily selecting the set of nodes based on the compression ratio. It then tries to maximize the number of compressible clusters so as to determine the suitable transmission mode for each cluster using a mode threshold that is a function of the number of nodes and the number of hops from a cluster-head to a sink.

To reduce the number of sensors involved in each CS measurement, Wu et al. [71] propose a sparsest random scheduling scheme for compressive data gathering in large-scale wireless sensor networks (WSNs), leveraging the spatial-temporal properties in the sensory data. The central theme of this study is that the measurement matrix is designed based on the representation basis and sensory data and according to the sensor network requirements rather than the network environment. By combining compressed sensing and network coding in the data-gathering scheme, Yin et al. [72] introduce a multi-hop topology in which the sink node adaptively adjusts the measurement formation according to the reconstruction of received measurements at each data-gathering epoch. The sink node dictates the data aggregation performed to balance the energy consumption among sensor nodes.

Xu et al. [73] exploit the CS paradigm for network tomography. Specifically, Ref. [73] leverages the fact that, typically, only a small fraction of network entities such as links or nodes are responsible for anomalies or degradation in network performance, as a limited number of congested links can be responsible for significant delays or high packet drop rates, and suggests utilizing CS theory in order to identify these few entities based on end-to-end measurements. Zheng et al. [74] provide an analysis of the capacity and delay of data gathering with compressive sensing in wireless sensor networks. The paper considers a random topology where sensor nodes are randomly deployed in a region, for both single-sink and multi-sink scenarios, and characterizes the capacity and delay performance improvement that the CS paradigm can achieve for data gathering. In particular, for the single sink, a simple routing scheme for data gathering with CS is suggested, and a tight capacity in the order sense is presented. In particular, the suggested routing scheme with pipelining scheduling algorithm for data gathering shows that the proposed single-sink scheme can achieve a capacity gain of $\Theta (\frac{n}{M})$ over the baseline transmission scheme, and the delay can be reduced by a factor of $\Theta (\frac{\sqrt{nlogn}}{M})$, where *M* is the number of random projections required for reconstructing a snapshot, and *n* is the number of randomly deployed nodes. For the multi-sink case, their architecture shows that the per-session capacity of data gathering with CS is $\Theta (\frac{n\sqrt{n}W}{Mn_{d}\sqrt{n_{s}logn}})$, and the per-session delay is $\Theta (M\sqrt{\frac{n}{logn}})$, where *W* is the data rate. The number of sinks present in the network is $n_{d}$, and the number of randomly selected source nodes is $n_{s}$. They validate the theoretical results with simulations for the scaling laws of the capacity in both single-sink and multi-sink networks.

## 4. Medium Access Control (MAC)

**Figure 3:**
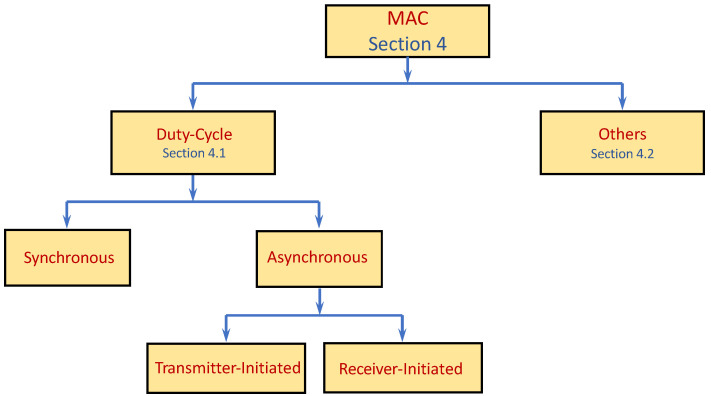
Schematic description highlighting the main MAC classes covered in the section.

Next, we move to the Data-Link layer and specifically to the medium access control (MAC) mechanism, which highly affects the performance of data gathering protocols, as it influences several performance aspects, such as reliability, latency, channel utilization, power utilization (which impact the lifespan of a sensor in particular and the network in general), etc. Even though many access protocols were suggested over the years for shared-channel networks, wired and wireless, due to their unique properties and requirements, extensive research has been conducted on MAC protocols targeted to WSN. Many WSN MAC protocols were designed to comply with various traffic patterns. Accordingly, in the sequel, we provide a short WSN-MAC protocol overview for ones that can support yet are not necessarily particular to data-gathering. We review a fraction of the vast literature on the topic and the numerous MAC protocols devised over the years. Figure 3 depicts a schematic partition of the papers discussed throughout this section according to the main MAC classes.

Energy consumption is one of the main concerns of WSN, and as previously explained, it needs to be considered in the design of protocols and algorithms in all the layers of the protocol stack. The highest energy consumer of a sensor or an IoT device is its transceiver, which consumes energy regardless of whether it is transmitting or only awake listening to ongoing traffic (e.g., [75]). One of the more prevalent solutions to save power is to embrace a duty-cycle mechanism in which the device is asleep most of the time (its transceiver is in low power mode), and is awake for transmitting or receiving data only a small fraction of the time. Another important aspect of wireless sensor networks (WSN) is channel utilization, that is, in a typical WSN, when multiple devices are trying to send reports simultaneously, air time is a crucial network resource. Particularly when the network is dense with multiple devices in the same neighborhood, issues such as coordinating between the users to avoid collisions and preventing users from occupying the channel for long time intervals are fundamental. Note that such issues can highly affect the performance in a dense network topology even when reports are not frequent. Accordingly, utilizing the channel (air time) efficiently is a crucial component for any such system’s operation and performance. These two highly vital aspects justify the significance given to the design of medium access control (MAC) protocols that are particular to WSNs. Numerous protocols have been suggested to cope with different WSN objectives and demands (e.g., [76,77]). This paper focuses on MAC protocols for WSNs, particularly on duty-cycled-based ones.

### 4.1. Duty-Cycle MAC Protocols

Traditionally, duty-cycle MAC protocols can be classified as either synchronous or asynchronous. In synchronous protocols the awake time interval is synchronized such that all devices are awake (or asleep) at the same time intervals (e.g., S-MAC [78], T-MAC [79]). Since the awake time intervals are quite limited, they can be highly congested and prone to collisions. Several synchronous protocols have devised mechanisms to attenuate this congestion and to allow more devices to transmit in each cycle, for example, DW-MAC [80], which allocates the awake time for transmission reservations that will be executed during the sleep interval.

In asynchronous protocols, each device has its own wake-up schedule. Accordingly, the main challenge is setting a rendezvous time when both the sender and the receiver are awake and devising a signaling mechanism that informs both that they are awake and can communicate. Asynchronous MAC protocols can further be divided into two categories, transmitter- or receiver-initiated. In transmitter-initiated protocols, the transmitter initiates the transmission by capturing the channel waiting for its designated receiver to wake up. For instance, in B-MAC [81], the transmitter transmits a long preamble capturing the channel prior to the data transmission, waiting for its intended receiver to wake up and reply. In X-MAC [82], the transmitter transmits a sequence of short preambles allowing the intended receiver to interrupt, notifying the receiver that it is awake. In WiseMAC [83], the transmitter learns the wake-up time of its intended receiver and starts the preamble transmission just prior to this wake-up time.

The second approach, the receiver-initiated paradigm, relies on the receiver, whenever awake and ready to receive data, to initiate the data exchange. The basic receiver-initiated MAC concept was introduced in RI-MAC [84], in which whenever the receiver wakes up, it transmits a predefined preamble, signaling to its potential transmitters that it is awake and ready to receive data. Several protocols took up the RI-MAC paradigm and suggested modifications and enhancements. Some protocols strived to reduce the energy consumed while a sender stays awake, waiting for its intended receiver to wake up. For instance, PW-MAC [85] and AP-MAC [86] suggested that each transmitter will learn its receiver’s expected wake-up time, and instead of staying awake waiting for its designated receiver to wake up, will wake up just before its intended receiver’s wake-up instance.

Another receiver-initiated enhancement is suggested by A-MAC [87], which aims to reduce the time in which a receiver and, consequently, its potential transmitters stay awake, by trying to determine whether there are pending packets for transmission and if it needs to stay awake or if it can go back to sleep after probing the channel. The enhancement relies on an additional frame, termed “*auto-ack*”, sent by pending transmitters, which follows the receiver’s probe packet and proceeds with the data transmission. The “*auto-ack*” frame is such that the receiver can decode a superposition of several such frames and determine whether there is traffic being sent. Even though the energy saved per cycle is negligible, the cumulative savings per day can be significant due to the numerous times a device wakes up probing the channel. RIVER-MAC [88] suggests two enhancements, one aiming at reducing the awake time a sender is waiting for its intended receiver to wake up, and one that aims at improving the RI-MAC collision resolution mechanism by letting an active receiver keep controlling the channel after invoking the collision resolution mechanism, and specifically during the silent backoff interval. MAR-RiMAC [89] suggested an amendment to the receiver-initiated approach, and in particular RI-MAC, to cope with the perpetual collisions, common in dense networks and heavy traffic loads, in which many devices are trying to transmit to the same entity (sink or relay). MAR-RiMAC relies on a reservation-based mechanism in which the reservations are short signals that can be transmitted simultaneously. After the designated receiver decodes the identity of the devices, it sends a transmission request and polls them sequentially, with no idle intervals.

As mentioned in Section 2, relying on EH requires adaptations that typically relate to the harvested energy source. For example, how to balance the harvested energy and the energy consumption can be a crucial factor in whether or not a scheme or protocol can be adopted by a network that relies on EH, and can be the primary factor impacting their performance. Adaptation requirements to support EH-based sensors span the whole network stack, including the MAC sublayer. Adaptation of receiver-initiated duty-cycle MAC protocol for energy-harvesting-powered wireless sensor networks, in which besides the usual MAC challenges, both transmitter and receiver need to have sufficient power for successful transmission, is given by Liu et al. [90].

### 4.2. MAC Protocols for Other Setups

Next, we review some MAC protocols and MAC adaptations for various setups, such as multi-channel, multi-radio, busy-tone utilization, or different from the duty-cycle approach. DURI-MAC [91] adopts the traditional busy-tone scheme and allocates a sub-channel for control such that while receiving data on the data channel, a busy signal is transmitted on the control channel, which notifies neighboring nodes of the ongoing transmission, and therefore helps avoid interference from hidden terminals. EM-MAC [92] utilizes multiple orthogonal radio channels and allows devices to dynamically select the channels for their transmissions based on the channel conditions they sense without the utilization of a control channel. Accordingly, EM-MAC can avoid using channels that are currently heavily loaded, inferior due to interference, or jammed. Typically, the traffic load sent by a node is spatially and temporally variable. Different nodes need to send different traffic loads due to their tasks, topological location, and the amount of traffic they need to relay. Furthermore, the same node can experience different loads at different time intervals due to events or requests triggering different traffic loads. Accordingly, several studies have explored an adaptive duty-cycle approach. For example, Ye and Zhang [93] have developed a reinforcement-learning-based self-adaptive sleep/wake-up scheduling approach. In the proposed method, each node (device) divides the time into time-slots, which are not necessarily synchronized between neighboring nodes. Each node decides whether to sleep or wake up in each time slot, and while awake, it decides whether to listen or transmit. The decision is based on its current situation and its estimation of its neighbors’ situations and is attained via Q-learning.

Gamm et al. [94] devise an alternative approach to duty-cycle, which utilizes two radios: the primary radio transceiver and an additional wake-up radio (WuR). The wake-up radio is a low-power receiver triggered by an external event and can turn on the main transceiver when required. Oller et al. [95] provide a detailed characterization of a specific WuR, the SubCarrier Modulation WuR (SCM-WuR), through physical experiments and measurements, evaluating it for different performance metrics and comparing it to other wake-up radio-based systems. The authors of [95] model and simulate their own designed WuR hardware platform, which is compared to four widely employed MAC protocols for WSN under three real-world network deployments [96]. Spenza et al. [97] further design and prototype a very-low-power-consumption (<1.3 μW), high-sensitivity (up to −55 dBm), fast-reactivity (wake-up time of 130 μs), and selective-addressing wake-up receiver (WRx) and describe its integration to a wireless sensor node. The authors leverage their WRx and present ALBA-WUR, a cross-layer solution for data gathering in wireless sensing systems. Similar to duty-cycled MAC protocols, wake-up radio-based protocols also distinguish between transmitter-initiated WuR (TI-WuR) protocols in which the transmitter wakes up its potential receivers (e.g., [98,99]) vs. receiver-initiated WuR (RI-WuR) that adopt the RI-MAC paradigm such that when a receiving node is ready to collect data, it wakes up all the nodes in its neighborhood by broadcasting a wake-up call (e.g., [100,101]).

An energy-harvesting-based MAC protocol for cognitive radio networks (CRNs) is suggested by Hawa et al. [102], in which the secondary users (SUs) utilize the transmissions by primary users (PUs) to harvest energy. Accordingly, the suggested protocol interlaces SUs’ data transmissions within these PUs’ transmission holes. The proposed energy-harvesting/data-transmission schedule considers the imbalance between the small amount of energy collected per PU’s transmission and the energy required by an SU data transmission.

Next, we mention several WSN MAC protocols that were specially designed for particular data-gathering setups in WSN and IoT networks, exploiting the special associated attributes (e.g., that the traffic patterns are always from sensors to the sink, or that there exists a set of predefined messages that need to be sent). Cohen et al. [103] design and analyze a data collection protocol based on information theoretic principles. In the suggested protocol, each sensor needs to convey one out of a bank of known messages to a sink. The protocol assumes a large population of sensors and devises a scheme in which a sink (or relay) can simultaneously collect messages from up to K sensors, without knowing in advance which sensors will transmit, and without requiring any synchronization, coordination, or management overhead. D-3 [104] exploits the fact that the traffic in data-gathering applications flows in a certain direction (toward a single or multiple sinks) to devise a wake-up that can significantly reduce end-to-end delay. Specifically, D-3 lays out the awake schedule of communicating nodes such that a packet can be forwarded toward its destination sequentially, without the need for a node to wait for its next-hop relay to wake up (i.e., the wake-up schedule is such that a relay wakes up in time to receive a packet just received by its predecessor).

## 5. Routing for Data Gathering in WSN

**Figure 4:**
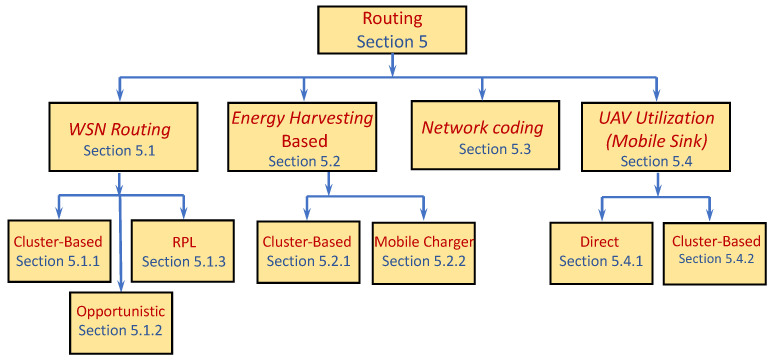
Schematic partition of the papers discussed in this section into the main routing topics covered in the section.

We keep climbing the layers, and in this section we address issues related to the Network layer. We start with a short review of WSN routing protocols. We note that routing-related aspects were also referred to in other sections of this survey. We mainly focus on the prominent and more recent protocols. We do not provide a comprehensive review of routing protocols in multihop WSNs and mainly explore routing protocols suitable for data gathering. Figure 4 provides a schematic distribution of the discussed papers into main topics. As with the schematic partition in the other sections, in order not to have too many, the topics are chosen such that the theme on each one encompasses several papers. The papers’ partition is rough: some papers can appear in more than one topic while others are only related to the topic.

We start with general WSN settings and then continue with routing protocols under energy harvesting constraints. Then we explore the utilization of network coding for data gathering, which leverages the multi-hop routing, allowing relays (intermediate nodes) to code the incoming packets before forwarding them toward the sink. We conclude this section by examining a different paradigm. Rather than utilizing the traditional approach of forwarding the sensed data via multiple relay nodes before reaching the sink, this paradigm relies on a Mobile Sink (MS) that traverses the network and collects the sensed data from the sensors it passes through.

### 5.1. Common WSN Routing Protocols for Data Gathering

The common setup for a data collection network comprises a set of devices (e.g., sensors) and one or multiple sinks that collect the reports. In many such scenarios, the sensors are unevenly dispersed over the terrain and in some cases can be mobile, can have a different distance to the sink(s), and the data needs to traverse multiple hops before reaching the sink, in which commonly the sensors themselves serve as relays toward the sink. Consequently, the performance experienced by different sensors (e.g., energy consumption, latency, reliability) can be markedly diverse. Various solutions, including energy-aware routing, compressed sensing, efficient MAC novelties and architectural innovations, have been suggested to try to improve the overall performance and to balance its variability; many are scattered throughout this survey under different subjects (e.g., Section 3, Section 4 and Section 6). In the sequel, we provide a brief review of routing protocols for WSN in general and data gathering in particular.

#### 5.1.1. Cluster-Based Routing Protocols

A significant milestone in WSN routing protocols is the low-energy adaptive clustering hierarchy (LEACH) protocol [105]. The basic LEACH protocol is an adaptive clustering-based protocol that dynamically selects sensor nodes as cluster-heads. Each cluster-head aggregates data from its cluster members and relays it to the sink. In order to distribute the high energy consumption imposed on cluster-heads between all the sensor nodes, the cluster-heads are dynamically selected according to a predefined probability that depends on the number of desired clusters. The resulting protocol causes continuous clustering hierarchy reelection, which facilitates energy balancing. The original version presented by Heinzelman et al. [105] considers a setup in which all sensor nodes can directly communicate with the sink; hence, each cluster-head can directly relay the collected information from its cluster members to the sink. However, as described in the paper, LEACH can be easily extended to a hierarchical cluster setup in which the cluster-head nodes of each tier are also organized in clusters such that each cluster-head relays its aggregated data to its higher-layer cluster-head, and so on up to the top layer of the hierarchy, at which point the data are sent to the sink.

Since its publication in 2000, numerous protocols have relied on LEACH’s clustering paradigm, suggesting enhancements and improvements for various setups and requirements. For instance, EE-LEACH [106,107] aims at improving the energy efficiency of LEACH by considering the sensor’s residual energy throughout the stages of the protocol. Specifically, EE-LEACH assumes that node deployment is implemented according to a two-dimensional Gaussian distribution. It forms clusters and selects their respective cluster-heads based on the residual energy of neighboring nodes. The relay nodes that forward the data aggregated by cluster-heads to the sink are selected based on their residual energy. A clustering procedure based on recursive rectangular partitioning of the network grid following the *k*-d tree algorithm is demonstrated by Anzola et al. [108]. The authors adjust a protocol that combines their clustering methods and report that it performs better than LEACH. Several surveys have summarized the successors of the LEACH protocol (e.g., [107]).

PEGASIS [109] was designed to address the overhead resulting from the cluster formation in LEACH. Specifically, PEGASIS replaces the clusters with a node-chain in which each node receives the data from its predecessor and transmits it to its successor in the chain. The data is gathered while getting fused along the chain until eventually, a designated node transmits it to the sink. PEGASIS relies on nodes having global knowledge of the network and shows that a simple greedy algorithm for the forwarding chain construction, in which nodes select their closest neighbors as the next hops in the chain, is sufficient to significantly reduce energy consumption. Similar to LEACH, and in order to balance the energy depletion in the network, different nodes transmit the fused data to the sink on each data-gathering round. P-LEACH [110] offers a hybrid between LEACH and PEGASIS that relies on cluster formation where cluster-heads collect and forward traffic. Rather than forward the traffic directly to the sink, or create a hierarchical cluster setup in which cluster-heads are also grouped into clusters with another cluster-head, P-LEACH adopts chain-based forwarding such that the cluster-heads are arranged in a chain along which the collected data is forwarded, as suggested by PEGASIS.

Several LEACH enhancements have relied on bio-inspired algorithms. For example, Siew et al. [111] utilize adaptive particle swarm optimization (APSO). This widely used swarm intelligence method mimics swarming behavior in bird flocking and fish schooling to guide its members to search for globally optimal solutions for cluster-head location selection. Tam et al. [112] extend LEACH to a 3D setting by employing a method based on fuzzy clustering and particle swarm optimization (PSO). Cui et al. [113] suggest a variant of the bat algorithm, which simulates bat prey echolocation behavior, to optimize the cluster-head selection for LEACH protocol. A routing path selection using an ant colony optimization algorithm is presented by Jiang and Zheng [114]. Clustering by mimicking groups of yellow goatfish is discussed by Rodríguez et al. [115]. The authors claim that the presented meta-heuristic is more efficient in avoiding local minima. An extension of LEACH for an IoT-designated industrial environment is presented by Karunanithy and Velusamy [116]. This work provides uniform CH selection, uniform CH dispersion over the industrial grid of IoT-based sensors, and tree-based routing selection that promises to be more energy efficient than known counterparts. The energy exploitation is claimed to be equal among nodes.

Mehmood et al. [117] devise a dynamic-size cluster-based routing protocol for WSN comprising a large number of sensors that are spread over a large area (the paper suggests pollution monitoring as a candidate application). The primary objective of the presented scheme is to effectively select CHs to be responsible for the main communication with BS and additionally defined chief nodes (CNs). Specifically, the sensor topology is divided into groups, where CNs collect the updated energy indications of other sensors within a group. There are also border nodes responsible for communication between groups. If their energy value drops below a threshold, the CNs can be reelected. The candidates for CNs and CHs are provided by an artificial neural network (ANN), which takes as inputs remaining energy, neighboring node count, the amount of outstanding data, signal-to-noise ratio (SNR), distances between nodes, CHs, CNs and the BS, traffic load, and so on. The simulations show a better lifetime than other selected known protocols; hence, the scheme is a better fit for pollution monitoring. Clustering and routing for a wind turbine system monitored by WSN are introduced by Durairaj and Selvaraj [118]. The discussed environment is unique because sensors placed at wind turbines can have their energy replenished by the turbine itself; hence, these sensors are always assumed to be charged. However, the distances between turbines and the BS are too large, and the grid is augmented by ground sensors that relay the measurements. In many cases, these ground sensors have to act as CHs. The authors propose system partitioning and clustering methods that may be hierarchical to specifically address this scenario. An interesting algorithm that also employs partitioning, by hierarchical grouping of sensors based on early knowledge of geographical transmission patterns in mobile WSN, is presented by Shifrin and Cidon [119].

#### 5.1.2. Opportunistic Routing

The opportunistic routing approach, which was designed for wireless networks, dynamically chooses paths toward the destination on a per-transmission basis, Biswas and Morris [120], Ye and Hua [121]. Opportunistic routing exploits the broadcast nature of wireless communication jointly with the spatial diversity of distributed nodes in a given route such that multiple nodes overhear each packet transmitted by a node. The node that receives the packet successfully and can serve as the best relay toward the destination (e.g., closest to the destination) becomes the next transmitting node. Harnessing opportunistic routing to duty-cycle MAC protocols encounters several obstacles. In synchronous duty-cycle MAC protocols (Section 4), the short time duration in which all nodes are awake (and specifically, all the potential relays are awake and trying to forward the same packet) can lead to artificial congestion and poor wake-time utilization. In asynchronous duty-cycle MAC protocols, since not all nodes are awake simultaneously, the use of overhearing, which opportunistic routing relies on, is limited and requires adaptations such that the transmitter will transmit to multiple relays upon its wake-up (e.g., [122]) or delay its transmission, choosing its relay opportunistically based on channel condition (e.g., [123]).

In a dense WSN, even under asynchronous duty-cycle MAC protocol, several potential relays can be awake simultaneously, which poses additional challenges when utilizing opportunistic routing. In addition to the relay selection problem—whether a transmitter should wait for the best relay to wake up or compromise on a less preferable relay, reducing its awake period and how long it should wait—it also encounters the collision avoidance problem between simultaneously awake nodes. Liu et al. [124] suggest a slotted contention-based scheme in which, following a probe sent by the transmitter, the awakened potential relays contend and transmit feedback concerning the routing progress they can offer. The transmitter selects the best possible relay out of the ones that replied, taking into account not only the metric chosen to evaluate the different relays, but also the waiting time of the link-layer transmissions. Under a similar set-up of dense asynchronous duty-cycle MAC protocol with multiple potential relays awake simultaneously, Hawbani et al. [125] try to control the number of potential forwarders, which influences both the transmitter waiting time and the number of packet duplications. The suggested solution relies on a two-step mechanism. First, each transmitter determines a *candidate zone* such that all nodes within the *candidate zone* are potential forwarders. Second, the candidates within the *candidate zone* are prioritized based on a combination of metrics that considers residual energy, transmitting direction, distance, and link quality.

#### 5.1.3. Routing Protocol for Low-Power and Lossy Networks (RPL)

A routing protocol for low-power and lossy networks (RPL) is a routing protocol that was specifically designed for networks composed of constrained nodes, which are interconnected via unstable and lossy links with relatively low packet delivery rates and typically only support low data rates (hence, low-power and lossy networks (LLNs)). Specifically, RPL is a distance-vector proactive routing protocol designed for IPv6 low-power devices with limited energy, processing, and memory resources (Winter et al. [126]). RPL constructs a tree routing topology termed the destination-oriented directed acyclic graph (DODAG), rooted at one or more sink nodes. The routing tree (graph), along which the traffic traverses, is constructed according to an objective function (OF) that can utilize a set of metrics such as energy consumption, latency, and hop count. The most common ones are OF0, which finds the shortest path (the path with the minimal hop-count) to the sink (Thubert et al. [127]), and minimum rank with hysteresis objective function (MRHOF), which finds the routes that minimize the link cost associated with the routes (Gnawali and Levis [128]). The cost is defined as the latency metric allowing RPL to find stable minimum-latency paths from each node to the sink, or it can be associated with the expected transmission count (ETX) metric, which allows RPL to find the stable minimum-ETX paths from the nodes to the sink (the default metric). In order to achieve stability, MRHOF also ensures that a route is changed (a node exchanges its preferred parent in the routing tree) only if the cost of the improved route is better than the current route by at least a predefined threshold. RPL is the de-facto IPv6-based routing protocol for the IoT. Accordingly, several OFs and possible enhancements have emerged during recent years, and several performance evaluations and comparisons have been presented. In the following, we discuss some of these OFs.

Abdel Hakeem et al. [129] analyze the performance of RPL in collecting smart meter readings over smart grid (SG) networks via Java-based simulations and IoT-LAB testbed experiments. Specifically, Ref. [129] evaluates the RPL performance under the two prominent objective functions Hop Count and ETX, in terms of packet delivery ratio, network latency, control traffic overhead, and power consumption. Barnawi et al. [130] utilize the Cooja network simulator to examine the performance of RPL under duty-cycle MAC protocol. Specifically, Ref. [130] simulates RPL over the classical XMAC protocol and its derivative ContikiMac, where, rather than using a long preamble waiting for the receiver to wake up, the sender repeatedly sends the same packet until a link layer acknowledgment is received. As a baseline, Ref. [130] uses NullRDC, an always-awake node. As expected, the results show that NullRDC is better in terms of latency, while ContikiMac outperforms the others in terms of power consumption. Al-Shargabi and Aleswid [131] also utilize the Cooja network simulator to evaluate which OF is more suitable for a WSN in healthcare scenarios. OF0 and ETX are examined in various network topologies, such as the grid and random topology, under diverse densities. They conclude that the OF0 is more efficient with respect to packet delivery ratio (PDR) and power consumption in the random topology setup.

Sousa et al. [132] propose an energy-efficient and path-reliability-aware objective function (ERAOF). The OF suggested by ERAOF linearly combines energy consumption and link quality (in terms of ETX) routing metrics. Even though the selected routes are not optimal in either one of the objective metrics, they provide a balance between energy efficiency and reliability. Rafea and Kadhim [133] suggest an energy threshold RPL (ETRPL), which, in addition to the ETX metric, incorporates in its objective function the remaining energy of the preferred forwarding (parent) node. ETRPL performance is evaluated via Cooja simulator. Sharma et al. [134] suggest another MRHOF-based objective function that takes into account three routing metrics: ETX, energy, and delay. Energy consumption, in order to increase the lifespan of the network, is also considered by Sankar et al. [135], which suggests cluster-tree-based routing protocol to maximize the lifetime of IoT (CT-RPL). As the name suggests, CT-RPL is a cluster-based routing protocol that involves three processes: cluster formation, cluster-head selection, and route establishment. CT-RPL first scans the nodes and group (cluster) nodes whose Euclidean distance from their centroid point is bounded, adding one node at a time. Next, each cluster selects its cluster-head (CH), utilizing a game-theoretic approach in which the node with the maximum payoff “*p*” value—which considers parameters such as residual energy, sensing energy, receiving energy, aggregation energy, and transmission energy—is selected as the CH node for each round. Finally, the route is established using the metrics residual energy ratio (RER), queue utilization (QU), and expected transmission count (ETX).

Another RPL enhancement termed weighted random forward RPL (WRF-RPL) is proposed by Acevedo et al. [136]. WRF-RPL suggests a load balancing over RPL mechanism, which distributes the traffic between multiple transmission paths, trying to avoid one preferred parent’s congestions. WRF-RPL OF relies on the composition of two metrics, the remaining energy and the count of parent nodes, where the latter aims at prioritizing parent nodes with more optional paths to the destination. Forwarding decisions are probabilistic according to the defined metric, such that nodes with a higher number of parents or hops are more likely to be selected than others. The authors utilize the Cooja simulator for evaluation. Rojas et al. [137] leverage a wired data center network labeling protocol to suggest IoTorii, a routing protocol for LLNs. IoTorii supports multiple paths between sender and receiver, and requires fewer table entries and control messages, with similar performance compared to the standard RPL.

Molnár [138] provides a graph-theoretic solution to the general problem of QoS-constrained routing in WSN that relies on RPL. The authors stress the difference between multi-objective optimization and multi-constrained problem setting. Vera-Pérez et al. [139] examine the integration of RPL to IEEE 802.15.4e with time-slotted channel-hopping (TSCH) medium access mechanism. In particular, Ref. [139] characterizes the long deployment delays required for such networks to become operational and able to start exchanging data messages. The article proposes an analytical model that estimates the average time that the synchronization process can take for a new node to join a TSCH-based network, as well as an estimation of the maximum time required for the formation of a complete network of this kind, and the additional time required to set the RPL-based routes. The paper validates the analytical model via simulations. A recent comprehensive survey on routing protocols for LLN networks in IoT (not exclusive to RPL) can be found in [140].

### 5.2. Data Aggregation Routing Protocols for Energy Harvesting WSN

As discussed in Section 2, an alternative for relying on batteries as the source of energy, with their imposed constraints (e.g., size, replacement, etc.), is to embrace energy-harvesting (EH) technology. However, as mentioned earlier, relying on EH imposes different constraints and limitations. Such constraints can make battery-reliant schemes impractical when devising data collection procedures. When battery-reliant schemes are applied to EH-based platforms, performance can be highly degraded. Specifically, relying on EH can induce high diversity between the different nodes, as different nodes can have different attributes, such as energy depletion and charging rates, which affect the nodes’ availability; in addition, different nodes can have different roles, such as different reporting rates or different report importance, requiring more energy usage, which eventually also affects the nodes’ availability. When a sensor is expected to transmit or receive a report, it needs to have sufficient energy to complete the transaction; therefore, reliance on EH needs to be considered when designing a scheduling protocol. For example, a data collection scheme that relies on EH should adapt the report rate to energy availability; it can compromise the rate of less important reports coming from the sensors to leave them sufficient energy for emergency reports. It can prioritize sensors with more energy over those with lower energy, especially when there is some redundancy in the reports received by different sensors. This is especially so when dealing with multi-hop routing, where many of the nodes serve as relays and have the burden to stay awake longer, to receive and transmit more, which escalates the heterogeneity of the nodes and accentuates the difference in importance between the different nodes. Furthermore, if on a single-hop network, we could rely on the receiver (sink) to have unlimited power (connected to a power source), in multi-hop topologies also, the receivers rely on EH; hence, when scheduling a transmission, we need to ensure that both transmitter and receiver have sufficient power to complete the transaction. In the following, we present several routing protocol adaptations for EH-WSN in the context of data aggregation.

The typical setup considers that each node (sensor) encompasses an energy harvester and an energy storage device and is solely powered by the renewable energy available to it by its energy storage device. A multi-hop topology is considered such that the data from many of the sensors need to traverse multiple links before reaching the sink. Jeong et al. [141] propose an adaptive data aggregation scheme for energy-harvesting WSNs. The suggested scheme relies on two residual-energy thresholds, lower and upper. Each node periodically estimates its residual energy level to determine whether or not to transmit data. When the node’s residual energy is either above the upper threshold or below the lower threshold, the node transmits its aggregated data. If its energy is below the lower threshold, the node enters energy-saving mode after transmitting the data. Its radio is turned off, and it waits to regain sufficient energy before turning its radio back on. In normal operation mode, where the residual energy is between the two thresholds, the node only aggregates data received from other nodes and collects its own sensed data. If a node’s aggregated data in normal mode exceeds its storage limit, the node transmits the data regardless of its residual energy. While this scheme is clear and straightforward to implement, it lacks latency evaluation and a discussion about rendezvous between a transmitter and a receiver.

Chen et al. [142] experimentally show that in energy-harvesting-based wireless sensor networks (EH-WSNs), the required nodes’ charging time to be ready to receive or send a packet is much greater than the time required for the contention resolution mechanism and dominates the data aggregation latency. In addition to the common collision definitions, the paper defines an “energy-collision”, which occurs due to battery level constraint and not due to simultaneous transmissions. Specifically, energy-collision occurs when a transmitter-receiver tuple is scheduled for transmission, but the transmission cannot take place because at least one of the two nodes has insufficient energy to transmit or receive the packet due to recent activity (insufficient time has elapsed since its last transmission or reception to harvest enough energy for the subsequent scheduled transmission). An adaptable data aggregation tree is constructed, which considers each node’s residual and harvested energy, and three energy-collision-aware data aggregation algorithms are proposed.

#### 5.2.1. Cluster-Based Routing Protocol That Relies on EH

Several studies have suggested various adaptations for LEACH WSN cluster-based routing protocol (e.g., [106]) for EH-based WSN (EH-WSN). Recall that LEACH’s cluster-head selection mechanism randomly selects sensor nodes as cluster-heads to distribute the energy consumption between them evenly, which can seemingly cope with the EH constraints. However, note that even when the nodes’ platform is exactly the same, the potential of different nodes to harvest energy, dependent on their specific ambient conditions, can be very different. Furthermore, the node’s location with respect to the sink can highly influence the amount of data it needs to relay toward the sink, causing high discrepancies between the energy utilized by various nodes. To cope with these discrepancies, Xiao et al. [143] modify LEACH’s cluster-head selection mechanism and define a new metric termed “energy potential function” to measure each node’s capability to harvest energy. The paper devises a cluster-head selection strategy that prioritizes nodes with higher expected stored energy (based on the currently available energy and their potential energy) to become cluster-heads, regardless of the number of instances that the node was selected as cluster-head in the past.

To address the imbalance between the energy expected to be consumed by cluster-heads (CHs) that are closer to the sink and are expected to spend more energy on relaying packets from farther clusters, and CHs that are further from the sink and expected to relay less traffic, Wu et al. [144] suggest an unbalanced clustering mechanism. In particular, cluster sizes are determined according to the distance (hop count) to the sink to balance the energy consumption of the CHs. Accordingly, clusters closer to the sink, which are expected to relay more inter-cluster traffic, will be smaller, so that they collect less intra-cluster traffic; clusters further away from the sink and expected to relay less inter-cluster traffic will comprise more nodes, so that they collect more intra-cluster traffic. The mechanism suggested to attain this balance is partitioning the network conceptually into concentric rings around the sink with linearly increasing radii. Each ring comprises nodes with the same hop distance to the sink. Clusters within the same ring will have the same size. CH selection is designed to balance the loads of each ring considering the available nodes’ energy, which is evaluated based on the EH rate. Following a similar approach, Yang et al. [145] assume a highly symmetric circular sensor network in which the sink (BS) is located at the center, and the sensors are distributed evenly in a disc around it. As with [144], the sensor field is divided into concentric rings; however, in this study, the rings are determined to have an equal area such that the number of sensors in each ring is expected to be the same. Under the given model, Ref. [145] analyzes the energy consumption of intra- and inter-cluster data transmission and derives the energy neutrality constraints, which guarantee that each node consumes less energy than the amount of energy it has harvested. The authors further devise a constraint formula of the number of clusters required in each layer (ring) that balances the average energy consumption of nodes in different layers. The energy neutrality constraints and the cluster parameters are used to obtain the parameters (number of rings, number of clusters in each ring, and minimum network data transmission cycle) that minimize the data transmission cycle. Based on the attained parameters, the cluster-based routing protocol is derived. The protocol consists of an initialization phase and repeated cycles divided into topology formation and data-gathering phases. Bahbahani and Alsusa [146] suggest two separate enhancements. The first, termed cooperative transmission strategy, enables nodes to serve as relays to relay undelivered packets from cluster members to CHs and from CHs to the sink node. The second mechanism, termed cluster-head duty-cycle, regulates the frequency at which a node can become a CH based on duty-cycling that adapts to the node’s energy-harvesting rate.

To conserve the energy of nodes that are more susceptible to energy depletion, Bozorgi et al. [147] select CHs, taking into account their residual energy, expected harvest energy, distance from the sink, number of neighbors, and, similar to LEACH, the number of times a node has already served as a CH in the past. The proposed approach, which combines centralized and distributed mechanisms, relies on a signal transmitted by the sink that can be received by all the nodes, which allows them to estimate their geographic distance from the sink. The network is partitioned by the sink into four layers based on distance. The sink further computes the individual coverage radius of each node (potential CH) based on distance (nodes closer to the sink, which are therefore expected to relay traffic coming from more distant clusters, will have a smaller radius, and thus less intra-cluster traffic; on the other hand, nodes farther away from the sink, which are therefore expected to have less inter-cluster traffic to relay, are assigned a longer radius, and therefore more intra-cluster traffic). The paper suggests a distributed contention-based mechanism for selecting the CHs in which the contention window takes into account the parameters mentioned above. Another cluster-based routing for EH-WSN is proposed by Ren and Yao [148]. The proposed routing scheme is divided into cluster establishment and data collection. It is suggested that besides the typical cluster members (CMs) and cluster-head (CH), a new entity be devised, termed in the paper scheduling node (SN), which is different from the CH. The main task of the SN is to monitor the energy of all the cluster members during the data collection stage and select a CH based on the monitored residual energy of the cluster members. The transmission range of nodes can also be adjusted based on their residual energy. The data collection stage, which adapts a round-based scheme similar to LEACH, is divided into the data transmission and CH selection stages.

Sinde et al. [149] aim to improve the network efficiency by three means: (i) clusterization mechanism that takes into account energy consumption during the data aggregation phase, (ii) duty cycle adaptation of each node such that each sensor node determines its mode of operation, and (iii) routing mechanism based on ant colony optimization that chooses the path between the source and the sink node that reduces the delay incurred. Overall, while the paper jointly addresses several topics in WSN, including those connected to energy-efficient data gathering, its main contribution is the detailed NS3 simulation.

#### 5.2.2. Mobile Charger

Today’s technology enables dedicated wireless charging equipment (WCE) to recharge the nodes’ batteries, prolonging the lifetime of wireless rechargeable sensor networks.

A model in which, in addition to the sink (base station), a mobile station will navigate through the WSN to collect data and charge sensor nodes is considered by Liu et al. [150]. The paper suggests a joint routing and charging strategy. The joint problem is decomposed into two sub-optimization problems: routing tree optimization and charging path optimization. Heuristic algorithms based on simulated annealing algorithms were applied to solve these sub-optimization problems.

A joint charging and routing algorithm with WCE-assisted data gathering is also suggested by Lu et al. [151]. The model suggested therein assumes that the BS (sink) can get the information of each node at any time, including node location, residual energy, and energy consumption rate. The suggested approach relates to data routing and energy supplement to undercharged nodes. The data routing algorithm considers several factors: sensor buffer occupancy, load, and energy. To find the route for the mobile recharging unit to traverse, they rely on Du [152] to seek the shortest Hamiltonian cycle between the nodes that urgently need energy replenishment traversed by the mobile recharging unit. Additional nodes along the traversed path can also be charged. Furthermore, the mobile recharging unit can also gather data from sensors with a critical buffer occupancy during its recharging cycle.

In the following section, we discuss network coding (NC) and, in particular, linear network coding in the context of data gathering in WSN.

### 5.3. Network Coding (NC)

Network coding leverages the routing protocols and the ability to construct multi-path routing between sources and their destination (the sink) to enable intermediate nodes to perform coding on the incoming packets before forwarding them. In the following section, we discuss network-coding-related works.

Linear network coding was first introduced by Celebiler and Stette [153] and evolved in the seminal paper by Ahlswede et al. [154] as a means to improve the network’s throughput, efficiency, and scalability, which can also be leveraged to improve the network resilience to attacks and eavesdropping. Linear network coding allows the network’s intermediate nodes (e.g., relays) to accumulate arriving messages and forward a newly encoded message, which is a linear combination of the accumulated packets, multiplying them by coefficients chosen from a finite field. The manner in which nodes encode and decode messages depends on the selected coding scheme. Network coding (NC) over wireless communication can reduce the number of transmissions by leveraging the fact that a single transmission is overheard by multiple nodes in the transmitter’s vicinity, and can therefore be utilized by each of these nodes, which will forward a coded packet with unique coefficients comprising its own message and the messages it overheard (e.g., [155]).

In WSNs, NC can be utilized for various traffic patterns, including data dissemination (one-to-many communication) and data gathering. Works on energy-efficient NC-based dissemination can be followed in the survey [156], with multi-hop routing being emphasized. In data dissemination in which the base station/sink distributes information to the sensor nodes, NC is beneficial mainly for distributing *control* messages (broadcast or multicast traffic) or, in case of unicast traffic, for recovering lost packets (retransmissions). The latter utilization relies on the fact that different nodes heard or did not hear different packets. Accordingly, nodes store packets they overheard, even if not destined to themselves. The transmitter (e.g., access-point) accumulates several packets that need to be retransmitted, each for a different receiver. It transmits a coded packet that is a composite of these accumulated packets. Each receiver can decode a missing packet by utilizing its stored overheard packets (e.g., [157,158,159,160,161,162], where the last two mainly focus on data dissemination of control management messages. It is noteworthy that Cohen et al. [157] also present a successful real HW radio deployment of their scheme. XOR-CoW [163] exploits the same concept to design an IoT protocol in which relays transmit coded packets that mix downlink and uplink traffic. Similar to other previously mentioned studies, the coding scheme by Swamy et al. [163] is over finite Galois field of size 2 (GF(2)) (i.e., XORing the coded packets).

Network coding is widely explored for data-gathering schemes. Typically, an NC-based protocol involves both the coding scheme and the multi-path routing. It relies on the relay nodes to overhear packets and perform the coding, and on the sink (or multiple sinks) to collect sufficient coded packets (combinations) to encode the sent information. The limited ability of the sensors constrains NC over WSN (e.g., limited storage to store overheard packets, limited computation power to perform sophisticated operations, limited awake time for packet overhearing, etc.). The utilized coding scheme influences the performance of the NC algorithm in several aspects such as the throughput, algorithm complexity, encoding complexity, decoding complexity, packet overhead (bits), required feedback, and so on. Note that many of these metrics are directly translated to air time and energy consumption, which must be considered in WSN. The random linear network coding (RLNC) encoding scheme (e.g., [164]) is widely used because, despite its simplicity, it can attain throughput that is close to the optimal one using a decentralized algorithm. In RLNC, relays transmit random linear combinations of the packets they receive, with randomly chosen coefficients from a finite Galois field (GF). The receiver must obtain a sufficient number of linearly independent combinations (packets) to decode the original packets. If the GF size is sufficiently large, the probability that the randomly generated combinations will be linearly independent is high. However, the receiver needs to know the coefficient used in each combination; hence, it needs to be sent as overhead piggybacked on each traversed packet. The larger the GF size, the higher the overhead. RLNC scheme with distributed encoding was utilized by Stefanović et al. [165] for a perimeter data-gathering objective, in which the data should reach the perimeter nodes that are located on the boundary of the covered area. The proposed scheme does not deploy routing algorithms or maintain routing information and relies on random walks.

One disadvantage of RLNC is its decoding complexity in the order of $O(n^{3})$, where *n* denotes the number of original packets. Sparse end-to-end erasure-correcting codes can reduce the decoding load on the receiver at the cost of introducing an additional, non-negligible delay. Feizi et al. [166] suggest a tunable sparse network coding (TSNC) scheme that tunes the level of sparsity as the transmission process evolves. This tuning process can reduce the delay overhead by using denser codes towards the end of the transmission while maintaining the complexity advantages of a sparse code. Prior et al. [167] propose two network coding schemes for information gathering, which are based on tunable sparse codes, with and without explicit feedback from the sink. The suggested schemes are designed for meter readings in a smart grid. Nistor et al. [168] further exploit RLNC and TSNC for data gathering and derived analytical bounds for a multi-hop line network using a fluid model, which is valid for any field size and various sparsity levels, and has two different feedback mechanisms.

SenseCode [169] adopts the NC paradigm, aspiring to balance energy efficiency and end-to-end packet error rate. SenseCode relies on nodes transmitting both uncoded and coded packets. Each node stores only a small portion of the packets it overhears, which can be attained by letting the node wake up sporadically within its duty cycle and stay awake for a short time interval each time it wakes up, storing overheard packets. Accordingly, each coded packet comprises only a small subset of the packets the node could have potentially overheard and coded. NetCoDer [170] concentrates on a star topology in which the star-head can be a sink or a relay that collects information from its neighbors. NetCoDer opportunistically selects, based on network conditions, a set of relays that, in addition to the data sent by the nodes, send additional coded packets with packets they overheard, which helps the star-head recover lost packets. To reduce the overhead, the relays use LNC coefficients based on the addresses of the sensor nodes. A similar idea that also does not rely on feedback was presented in SR-Code [171] in which nodes and relays send redundant coded messages to help the sink recover lost messages. SR-Code utilizes the XOR operator (GF(2)). Similar to NetCoDer, SR-Code reduces the overhead by using a bitmap to identify the coded packets rather than the address of the sending node. Al-Hawri et al. [172] assume a data-gathering setup with single or multiple gateways (sinks) that can collaborate (exchange information via accessible shared distributed storage system). The paper distinguishes between traditional relays, which forward packets they receive as-is, and encoding relays, which perform network-coding on the packets they receive before forwarding them. The authors suggest a mathematical model and a heuristic algorithm to determine the number of network-encoding nodes and their location, which is insufficient for the aggregate received data at the gateway/gateways to be decodable, taking into account link failure scenarios.

Protocols that utilize NC in WSN need to balance achieving the NC expedience and diverse performance criteria. On the one hand, the motivation is to send only coded packets, as sending uncoded packets degrades the NC gain. On the other hand, a strategy in which a relay waits for packets to arrive in order to code them can yield unacceptable delays. Furthermore, storing arriving packets while waiting for additional packets to arrive before coding them can result in a buffer overflow and packet loss. To overcome these drawbacks, Chen et al. [173] suggest an opportunistic network coding (ONC) approach in which the relay can transmit either coded or uncoded packets. Each relay determines whether to transmit a coded or uncoded packet according to its queue state at each transmission opportunity. For the simple topology of a relay interconnecting two nodes that communicate with one another, Ref. [173] presents an ONC strategy that can achieve the optimal delay/power tradeoff. Mirani et al. [174] adopt the ONC paradigm for data dissemination in vehicular ad hoc networks (VANETs), for which the mobile nodes (vehicles) employ a decode-and-forward scheme with subjective timers determined according to their distance from the source. In Tan et al. [175], an opportunistic routing protocol with opportunistic network coding is proposed for correlated data gathering.

Next, we provide more recent works that utilize NC in WSNs and related networks that are not particular to data-gathering applications. Marques et al. [176] propose to use NC in a fog computing scenario, since in the fog computing system architecture, the data measured on a node should be delivered to many other destination nodes. The authors design a protocol for encoding and decoding and provide a design to incorporate it on the MAC level. Uwitonze et al. [177] consider a setup in which a WSN has been split into multiple disjoint partitions and suggests a polynomial-time heuristic algorithm based on space network coding termed relay placement using space network coding (i.e., rather than send additional coded data, additional relay nodes are deployed) for finding the optimal number and positions to place relay nodes for restoring the network connectivity.

A network coding backpressure routing scheme for data aggregation in large-scale Internet of things (IoT) networks is explored by Malathy et al. [178]. The proposed routing scheme exploits network coding for the data aggregation process, which improves the throughput of the network by eliminating redundant packets. The paper relies on cluster-based routing that selects the cluster-heads (CH) based on the available energy and distance, which helps to minimize traffic congestion and provide efficient data transmission.

### 5.4. Data Collection Utilizing Mobile Sink and Unmanned Aerial Vehicle (UAV)

A different paradigm for the traditional setup of data gathering in WSN, which relies on a single or multiple static sink(s) towards which all traffic needs to travel, is the utilization of one or multiple mobile sinks (MSs) that traverse the terrain and collect the reports from the devices. Such mobile sink(s) can be the traffic’s final destination or just an accessory that collects the data and transmits it to the sink. Note that in the latter case, the final destination is not necessarily located within the wireless network realm and can be located outside it (e.g., within the Internet or the cloud). Since these mobile sinks are unmanned aerial vehicles in many cases, these systems are sometimes called unmanned aerial vehicle-wireless sensor networks (UAV–WSNs). This section reviews several state-of-the-art developments in mobile sink(s). Since sometimes the MS routing challenges are interleaved with the MAC layer’s challenges, the solution suggested in some papers, and accordingly their description, incorporated both layers.

Typically, data-gathering protocols that utilize moving sink(s) aim at optimizing some performance metrics, such as overall power consumption, average or worst latency of the data, trajectory traversed by the mobile sink, awake time of the sensors, maximizing the life cycle of the network, and so on. There are different options to classify these protocols: they can be classified according to this aforementioned performance objective, or other categories, such as characteristics of the moving sink, its speed and constraints, network model, communication standard utilized, and so on. In this section, we will classify the protocol into two classes: protocols that solely rely on the moving sink with no intra-traffic between the nodes (all the traffic is directly transmitted to the moving sink), and protocols that combine routing between the nodes and traffic forwarding to the sink. This latter class will mostly include cluster-based protocols in which the nodes are clustered and cluster-heads (CHs) are selected, but rather than the CHs routing the traffic towards the sink, they forward the traffic directly to the mobile sink.

#### 5.4.1. Routing Directly to the Mobile Sink(s) with No Intra-Node Data Forwarding

In this section, we review papers in which the only data traffic is between the nodes and the mobile sink, and no data is forwarded between the nodes.

Zhan et al. [179] consider a general fading channel model and suggest an efficient sub-optimal solution that minimizes the energy consumption of all sensor nodes (SNs) while ensuring that data is collected reliably from all SNs with bounded outage probability. The suggested solution decouples the joint optimization problem, which considers both the SNs’ wake-up schedule and the unmanned aerial vehicle (UAV’s) trajectory, into two separate optimization problems, ensuring that the amount of data collected from each SN reliably exceeds a threshold. One of the formulated optimization problems is non-convex due to non-convex constraints, and therefore it needs to be relaxed. The two problems are solved iteratively to obtain an approximate solution. A flight time minimization problem for a UAV that collects data from a set of energy-constrained ground sensors is studied by Gong et al. [180]. The sensors are assumed to be located on a line (one dimension). Each sensor has a certain amount of data to upload. The UAV can collect data either while flying or while hovering, and only from a single sensor at a time. Accordingly, the UAV’s trajectory is divided into non-overlapping data collection intervals, each dedicated to collecting data from a single sensor. The objective is to minimize the total flight time of the UAV from an initial point to a destination by jointly optimizing the division of intervals, the UAV’s speed, as well as the sensors’ transmission power. The flight time minimization is formulated as a dynamic programming (DP) problem, where each DP stage considers flight time minimization for a single-sensor data collection scenario. The algorithm for the single-sensor case is used to find the UAV’s optimal speed and the sensor’s transmission power. It is shown that the UAV’s optimal speed is proportional to the given energy of the sensors and the inter-sensor distance but is inversely proportional to the data upload requirements.

The metric addressed by Liu et al. [181] is the age of the information. In particular, Ref. [181] utilizes UAV, and suggests two age-optimal trajectories for it to collect the data from the ground SNs, one that minimizes the age of the ‘oldest’ sensed information among the sensors, and another that minimizes the average age of the information sensed by all sensors. It is shown that both age-optimal trajectories correspond to the shortest Hamiltonian path in the wireless sensor network, in which the distance between any two sensors is represented by the amount of inter-visit time. The authors adopt dynamic programming and genetic algorithm to find the two different age-optimal trajectories. Liu et al. [182] also utilize a UAV to collect the data from the ground sensors. The model suggested therein assumes that the sensors (nodes) are randomly distributed over a square area. The area is partitioned into small square cells. The UAV flying above the cells hovers above each cell to collect all the data of the sensors within the cell. The paper studies the amount of data per unit time that the UAV can collect from the ground nodes as a function of the number of cells, the height of the UAV, the number of sensors, and the energy capacity of the UAV. The paper suggests a similar analysis when multiple UAVs are utilized. It seeks the optimal number of cells to maximize the per-node capacity under the suggested model and shows that under the suggested data collection network, multiple UAVs can significantly improve the per-node capacity attained by a single UAV. The balanced network communication protocol (BNCP) that utilizes UAV as a mobile sink is suggested by Qin et al. [183]. There is no inter-sensor routing in BNCP, and all sensors’ communication energy is spent on the sensor-UAV transmission links. BNCP is implemented and evaluated.

#### 5.4.2. Cluster-Based Data Forwarding

Rather than the mobile sink node traversing the network between all the sensors, a different approach groups the sensors into clusters and selects cluster-heads (CH), which collect the data from all cluster members, thus decreasing the traverse of the mobile sink. In general, data gathering in a clustered WSN imposes a tradeoff between the energy consumed during data collection within each cluster and the energy consumed by the mobile sink. On the one hand, the higher the number of clusters (smaller clusters), the less energy is consumed during data collection by the cluster-heads (CH); on the other hand, it follows that the UAV will have to access more CHs on its route, and consequently, the energy spent by the UAV will be higher in that case. This trade-off is one of the main challenges in addressing the joint problem of clusterization and CH selection, jointly with finding the path traversed by the UAV, as reflected in several papers discussed below.

Najjar-Ghabel et al. [184] propose a two-phase algorithm, termed DGOB, for data gathering in WSNs in an environment impaired by obstacles, which utilizes a mobile sink that traverses the network and collects the data. Both phases, node clusterization and MS trajectory construction, exploit artificial intelligence (AI) schemes. Tazibt et al. [185] utilize a small-scale drone to gather the data from scattered sensors. Like in several other papers that rely on UAV to collect data from cluster-heads, the challenge in [185] is two-fold: (i) clusterization and CH selection (in contrast with other papers, this paper allows multi-hop clusters, such that cluster members are up to a predefined number of hops from their CH); and (ii) plan of the drone trajectory for traversing through all CHs with minimum energy consumption (e.g., minimum path length). Even though the two problems are related, the authors solve the two issues sequentially. They first solve the CH selection by formulating an optimization problem that seeks the minimal set of cluster-heads that guarantee that all nodes are at most *h* hops from a CH in the set. After determining the CH set via linear programing, they utilize the 2-opt heuristic, which relies on a simple local search algorithm for solving the traveling salesman problem, in order to find the optimal travel trajectory for the drone between the selected CHs.

Kumar and Dash [186] also suggest a data-gathering-by-mobile-sink scheme in WSN. The model in [186] assumes that the mobile sink is moving along a pre-specified path with constant speed and can collect data while traveling. The paper denotes all the sensors that are in transmission range from the mobile-sink traversed path, and can therefore relay traffic to it, as sub-sinks. All other nodes need to forward their data to these sub-sinks, possibly through multi-hop communication, in order for them to relay the data to the mobile sink. The paper suggests two different models: the first assumes that the mobile sink can receive data from only one sensor at a time, while the second assumes that the mobile sink can receive data from multiple sensors simultaneously. Both suggested algorithms comprise three phases: (i) identify the relay nodes (sub-sinks) that are within transmission range from the mobile sink trajectory (unit disk graph model), and partition the path into segments that are the union of all the transmission disks of all the sub-sinks; (ii) determine the communication time each sub-sink can have with the mobile sink, and accordingly, the amount of data it can transmit; (iii) utilize a network flow approach to determine which sub-sink transmits to the mobile sink in each of the mobile sink’s path segments. Ebrahimi et al. [187] aim to optimize total transmission power between WSN cluster-heads in an IoT network. The problem is split into subproblems, which include CH assignment, building of the forwarding tree within each cluster, and optimal UAV trajectory calculation. The data transmitted by CH to UAV is pre-processed by a specialized optimized compression. A genetic algorithm for energy-efficient CH selection is employed by Wu et al. [188].

The focus of Zahra et al. [189] is on MS that traverses a WSN that relies on a cluster-based data collection protocol in which cluster-heads are responsible for collecting and transmitting the cluster members’ sensed data to the MS. The paper examines a scenario in which the MS is constantly moving in a predefined trajectory, regardless of whether the data transfer was completed or not. Accordingly, when the cluster aggregate data is too large, the CH cannot complete the transaction. The authors suggest a mechanism in which, in case the CH cannot complete the transaction, it can use different cluster members as relays to continue the transaction after the MS has moved out of the transmission range of the CH. Likewise, Zhang et al. [190] also suggest a hybrid approach that combines the MS with hierarchical routing-based protocol relying on node clusterization. In order to improve the efficiency of the MS data collection, Ref. [190] suggests utilizing virtual heads (VHs) that lie on the MS trajectory on the cluster boundaries that also transmit collected data to the MS. The channel access relies on a random access mechanism.

The UAV can be of different physical structures. While some machines are able to slow down and even to hover, others, especially the winged UAVs, can only fly with a constant velocity. This limitation poses an additional challenge; see Say et al. [191] for a possible solution. To address the velocity limitation, the grid topology of the UAV’s coverage is divided into frames, and the most distant frames get the highest priority. The priority-based transmissions from sensors to the moving UAV are incorporated into a MAC layer, by introducing a priority-based contention window adjustment scheme. A smaller contention window is assigned to the frame where sensors send packets from the rear side of the UAV, and should therefore have a higher transmission priority. This results in a low packet loss when the UAV flies forward. On top of this architecture, a frame-selection-based routing protocol is formulated.

## 6. Wearables and Wireless Body Area Networks (WBAN)

**Figure 5:**
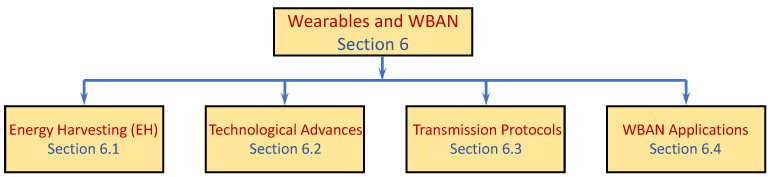
The main topics in the context of wearables covered in the section, and a rough partition of the papers covered by these topics.

Numerous applications rely on data gathering and report collection in WSN and IoT, and some are mentioned throughout the paper. There is no doubt that the essence of WSN is the applications that utilize the infrastructure discussed throughout this paper. Providing a thorough review of such applications can encompass several surveys. Since wearables are becoming highly popular and a ubiquitous application which imposes new challenges in the context of data gathering, we discuss several recent results and related challenges in the context of wearables. Figure 5 depicts the main topics discussed in this section.

Wearable technology refers to smart devices attached to the human body or apparel to monitor the user and their environment. Wearables are designed to detect, analyze, and transmit information, which allows continuous monitoring of the subject. In some cases (e.g., healthcare applications), feedback is returned to the wearer with strict performance constraints (e.g., reliability, latency bounds). Such applications can necessitate the monitoring of both bodily conditions, such as blood pressure, blood glucose level (e.g., in conjunction with an insulin pump), ECG, EMG, body temperature, accelerometer, and gyroscope, as well as environmental conditions that might influence the user, such as temperature, humidity, CO_2_ level, dust level, and location.

Sensing essential human physiological parameters led to the innovation of wireless body area networks (WBANs). WBAN typically relates to a small area network that spans the whole human body. It comprises devices (wearables) located in the apparel, on the body, or under the skin, and are wirelessly connected. Even though tracking physical conditions applies to diverse domains, including medical, social, and economic ones, each with its particular implications and extensive research in the field, the specific challenges of acquiring data in WBANs necessitated new solutions designed for WBAN. Specifically, since the sensors in WBANS are designed to be located close to the monitored individual, they impose particular challenges related to their specific structural, functional, and size-related constraints. In the sequel, we dwell on some of the most critical challenges and recent promising results on WBANS. The main objective of this survey is to understand the data-gathering challenges. In the special case of WBANS, we also elaborate on the sensors’ physical and mechanical structure, which directly impact the data acquisition process.

Many of the challenges pertaining to WSN in general, which are described throughout this survey, also apply to wearables. However, some of these challenges are exaggerated when applied to wearables due to their unique characteristics. For example, energy-related challenges that are essential to address and substantially influence any WSN design and performance, open a different perspective on wearables. Specifically, due to their sensing devices’ tiny size, the energy storing capacities are limited. Yet, many wearable applications, particularly healthcare ones, employ continuous sampling and communication tasks that constantly consume energy and deplete the device’s battery. Frequent battery replacement, which can be a burden in any WSN, can be even more cumbersome in wearables and can hinder the adoption of these technologies (e.g., when the wearables are implants, battery replacement can involve an invasive medical intervention). Accordingly, energy-efficient approaches designed for wearable sensor networks are important for disseminating the technology into additional domains. An inclusive review of energy-efficient approaches designed for human context recognition (HCR) based on wearable sensor networks is given by [192]. This paper classifies energy-efficient mechanisms for health-related HCR applications, based on the task that the mechanism is aimed at to reduce its energy consumption (e.g., sensing, communication, computation). The paper reviews the related works according to the classification.

### 6.1. Energy Harvesting (EH)

Energy harvesting as a battery alternative has been discussed in Section 5.2. However, EH in the context of wearables encounters new challenges, such as critical reliability level, expected tiny size and position in/on the human body, limited exposure to energy sources such as solar energy, and so on. Accordingly, we revisit EH and review some EH studies in the context of wearables. The three most prominent techniques include photovoltaic cells attached to a wearable (energy is accumulated from environmental illumination), thermoelectric nanogenerators (the energy source is the heat produced by the human body), and kinetic energy harvesters (energy is created by natural body motion). All energy harvesting cases pose a fundamental challenge of effective energy management, which involves continuous decision making regarding how much energy to spend on sensing, measurements, on-board classification, and transmission (see also Section 5.2).

We present examples for each of the techniques mentioned above in the context of wearables. Thermoelectric energy-harvesting units exploiting self-generated human heat are suggested to be fabricated straight into the textile of the garments [193]. Energy harvesting for activity-aware wearables is designed by Khalifa et al. [194]. The idea is to remove the accelerometers, which consume about 80% of the battery. Instead, the authors propose to employ kinetic energy harvesters, which will convert human motion into electrical power. Additionally, to compensate for the accelerometers’ removal, the input and output of the kinetic energy harvester would be analyzed by a specialized activity classifier to track and identify the human activity (i.e., to perform the primary task of the wearable). Light-based energy-harvesting wearables are discussed by Park et al. [195]. It is noteworthy that as the available luminosity can be highly unpredictable, the overall functioning, data-gathering, and transmission process would imply an optimization problem. Henceforth, the same authors suggest a protocol to optimize the number and accuracy of interpretation of human gestures by an energy-harvesting wearable device under an energy budget, Park et al. [196]. To this end, they constructed an analytical model that characterizes energy consumption based on experimental data and formulized the optimization problem. Esteves et al. [197] suggest incorporating energy harvesting as part of MAC 802.15.6. The proposed MAC modification includes the usage of some body sensors as relays. The managing part at a hub (e.g., a smartphone) sets the optimal relay charging times to perform the data transmission by relay effectively. A source and the relays send their energy-harvesting updates within the request for cooperation (RFC) packets. The charging times are calculated according to the amount of energy at the source and relays and are updated in accordance with previous packet transmission success or failure.

### 6.2. Technological Advances

WBAN utilization is penetrating new domains, spanning a wide variety of sensors, each providing a different aspect of the monitored subject. For example, healthcare applications are expected to provide a wide span of indicators from various physiological parameters. Specifically, to provide a comprehensive status of the examinee, it requires, in addition to the standard indicators such as heart rate and physical activity that many of us already have integrated in our watch, information at a deeper level and molecular level insight into the dynamics of the wearer.

To cope with the growing popularity of wearables and their expansion to a broader scope of applications, especially in healthcare systems, with the increasing demand to improve quality of service (QoS) and quality of experience (QoE), a new generation of wearables has emerged. This generation relies on several technological advances in both the device and cloud realms. The novel device technology utilizes new soft-sensing technologies, including innovative wearable materials such as conducting polymers, rigid forms of hydrogel, gold and silver nanowires (to create nanowired tattoos as stretchable sensors), carbon nanotubes, liquid metals, ionic liquids, and others. These materials give rise to novel sensor families, such as electro-physiological (acting on the electric potential difference) physical and chemical sensors. See the latest advances in the following related works [198,199,200]. For example, besides reviewing the key developments in sweat-sensing technology, Bariya et al. [198] examine the requirements of the underlying components embedded in sweat-based wearable sensors and discuss challenges for integrating wearable sweat sensors in the development of personalized healthcare. Sweat-sensing technology has been described earlier; see, for example, Salvo et al. [201], where a device containing two humidity sensors located at different heights from the skin is designed in a way that allows one to measure the sweat rate by the difference in readings of these two sensing sub-units. Thermal comfort control by calculating the relation between the vapor pressure and the temperature is designed into watch-type sweat sensors by Sim et al. [202]. Sweat measurement by sweat biomarkers (in particular, pH and Na^+^) is implemented by Song et al. [203]. In short, the biomarkers cause a change in electric potential near the measuring device, allowing for accurate sweat measurement during physical activity. The authors also note that the change in electrical potential allows for energy harvesting, which can be effectively managed to self-power the device and thus allow for a battery-free design. In general, the material imprinted into the working reacting electrode determines which substance (i.e., a chemical component present in the sweat) it will react with. For example, an application of electrochemical differential pulse voltammetry to sense the sweat to measure the level of caffeine is devised by Tai et al. [204].

The main challenge in the devices is to implement capabilities of effective data gathering, filtering, and transmission within microscopic computing units. Some biometric sensing (especially made by specific configurations of adjacent sensor sets) produces large amounts of data (as in ECG sensing) that should undergo an appropriate local density reduction. To ensure conformity with the sensing devices, paper lithium batteries are proposed (see, e.g., [205]). In addition, many sensors are configured only to transmit following special hazards. For example, ECG sensors should trigger an alarm in case the heartbeat is abnormal. This local data processing is a preliminary phase that facilitates the more intensive data analyses in the intermediate data collection unit (the smartphone), and finally, the destination server. To treat noisiness and variability (if the sensor sampling frequency is too high) of raw data from an accelerometer or gyroscope or loss of data (if the sensor sampling frequency is too low), variable sampling is proposed by Li et al. [206]. The offline ML classification algorithm is used to identify and predict the physical activity.

The recent development of miniature sensors combined with minimization of battery size and energy harvesting advances is sometimes referred to as a new paradigm, known as Wearables 2. For example, Ling et al. [207] provide a detailed description of various types of sensors, means of attaching them to the human body, multiple parameters the sensors are capable of measuring, and techniques to communicate and process the measured data.

### 6.3. Transmission Protocols

We note that while many of the works mentioned above employ a smartphone device with an application that is presumably tailored to receive data from the wearables, having a dedicated hardware device that would offload some of the networking burden (e.g., potential simplification of the packetizing process) of the sensors would be more effective and reliable. Indeed, in Pathak et al. [208], a central processing hub allows one to circumvent the cumbersome processes of sensor identification, sensor joining, and reconfiguration by providing a designated interface. The authors provide real hardware implementation, explore various performance metrics, and provide energy measurements.

Several available protocols for data transmission are suitable for WBAN. We mention here the low Bluetooth energy (BLE; see, e.g., Townsend et al. [209] for a detailed protocol stack description and Gomez et al. [210] for performance evaluation and comparison with ordinary Bluetooth and other protocols). For distances of several centimeters, near-field communication (NFC) protocol (see Coskun et al. [211] for the theory and Kim et al. [212] for a description of possible device designs and applications). Details of the IEEE 802.15.6 standard, which covers WBAN, can be found in, for example, Kwak et al. [213]. One sees therein how the human body communications PHY layer in particular is defined.

### 6.4. WBAN Applications

Even though, as stated earlier, there is no intention to provide a thorough review of WBAN applications, we mention several interesting ones. Monitoring of workers involved in extreme conditions or whose activity can be potentially dangerous (e.g., Lee et al. [214]) suggest wearable sensors for monitoring miners or construction professionals. This study evaluates integrated wearable sensors for measuring construction workers’ personal level of workload, individual factors, and physiological reactions during roofing activities. A fatigue detection system for car drivers by Chang et al. [215] includes smart glasses, which are able to identify possible drowsiness by an IR detector aimed at the driver’s eyes, equipped with BLE transmitter. A small processor embedded into the glasses gathers and pre-analyzes the data and then transmits them to the on-board computer in the car. The latter makes a decision about whether to issue a fatigue warning and sends it to the cloud. Wearables can be attached to a human body in order to sense the environment for the safety of the carrier, as in Wu et al. [216]. The data (humidity and temperature) is then transmitted to a mobile unit, where it can be analyzed locally or further transmitted to the cloud in order to issue timely warnings. Classification of sports activity is implemented and validated by experiments by Qi et al. [217], where the activity is identified from accelerometer and ECG measurements done by chest and wrist sensors. The measurements are transmitted to a smartphone, which performs the data processing. SVM is employed for the classification.

You et al. [218] suggest a real-time wireless body sensor networks (WBSNs) scheme for welfare assessment and disease monitoring, prevention, and treatment. The suggested scheme is composed of three components: sensing, communication, and management. Sensing attains a set of physiological parameters, such as heart rate, body temperature, ECG, temperature, blood pressure, blood glucose, heart rate, and oxygen saturation, from designated sensors embedded on a smart shirt worn by the monitored user. Communication handles the processes of delivering the sensed physiological data and controlling instructions to a backend server through wireless networks. The transmission protocols can be divided into two segments: the transfer of information from the sensors to a central terminal located at a smartphone and the transfer of information between the smartphone and the designated server, which is located on the healthcare cloud. The communication relies on multiple communication protocols including Bluetooth, WiFi, and 3G/4G (which can be replaced by 5G where available). Management is responsible for collecting, classifying, and monitoring the physiological data, and furthermore, being able to issue warning messages to medical professionals or caregivers whenever the physiological data are abnormal. Additional applications utilizing data gathering can be found in the following survey [219].

We conclude by emphasizing that the benefits of WBANs have not run their course yet. The development of data gathering will jointly progress with the ongoing advances in sensor construction and manufacturing capability, development, and standardization of Wearables 2 and beyond. Development of specialized post-processing platforms poses a specialized challenge, and the urge to make progress in this area is acute. To illustrate this, on account of an ever-growing population, specialized platforms for elderly care are needed; see, for example, the recent papers [220,221] about wearables designated for elderly patients and references therein.

Finally, wearables have been recently harnessed for combating the COVID-19 disease. Early identification of COVID-19 symptoms by evaluating the resting heart rate during the asymptomatic (presumably infectious) period and analysis by a deep learning framework is evaluated by Bogu and Snyder [222]. While the precision of such a tool is clearly inferior to the standard medical assays, it may be useful to provide a preliminary alert for the early onset of the disease for people in risk groups. Hassantabar et al. [223] suggest a framework termed CovidDeep that combines commercially available wearable physiological feature sensors (WMSs) and a simple yes/no questionnaire with efficient DNNs for pervasive large-scale monitoring of disease onset and health condition. The automatically extracted raw data and medical background and symptom responses are combined with synthetically generated data to train the DNN architecture. Grow-and-prune synthesis is used to generate accurate and computationally efficient models that can be deployed for COVID-19 inference.

Since viruses can spread between people who are in close contact with an infected person, and since infected people may be asymptomatic, the pandemic taught us that it is best to keep a safe distance from others (see, for example, Cortellessa et al. [224] for close-proximity risk assessment for COVID infection). Accordingly, most health institutions recommend keeping physical distance between people in public places (commonly termed as ”social distancing”) in order to stop the pandemic from spreading. Furthermore, people who were in the proximity of an infected person (tested positive for COVID) are encouraged to be examined, and several governmental regulations even require such people to stay in quarantine. Several recent studies have suggested leveraging wearables for contact tracing in order to identify the hazard from close proximity. For example, Ng et al. [225] focus on Bluetooth low energy and discuss the different data flow approaches and the accuracy of smartphone vs. smartwatch applications in proximity detection. Bian et al. [226] utilize wearables to monitor social distancing as recommended for preventing COVID-19 spread. In particular, the authors design compact potentially wearable oscillating-magnetic-field-based proximity-sensing prototype systems suitable for the relevant safety distance and able to track social distancing much more reliably than the current Bluetooth-based smartphone technology. Shubina et al. [227] provide a brief technical overview of the main contact-tracing approaches and the challenges they impose on wearable technology. The paper also provides a short overview of the existing solutions deployed for contact tracing and a discussion on the potential effect of wearables in tackling the spread of a highly contagious virus. More works from the past year discuss the use of wearables for remote management and automated assessment of COVID-19. Amft et al. [228] provide an overview of institutional initiatives and alternative, more accurate technologies for detection of infection symptoms and possible contact with infected individuals. Channa et al. [229] is a systematic review of the two categories of challenges: on-body sensors and their clinical utilization in screening and contact tracing.

## 7. Concluding Remarks

Data gathering in modern WSN and IoT networks encompasses many challenges, which span the entire communication stack. Many techniques, protocols, and solutions have been proposed over the years, but as technology advances, new challenges and new opportunities arise. In this survey, we reviewed these main challenges and opportunities, as well as recent advances, and related them to specific data-gathering research domains. We provided a comprehensive state-of-the-art data-gathering literature review in modern WSN networks, distinguishing between the communication layers and the research domain.

We first summarized general architectural novelties and emerging architectures. We reviewed several new technological advances and their influence on sensing device design, the platform carrying it, and the transceiver. We reviewed the effect of these architectural advantages on specific applications, such as agriculture, smart cities, smart homes. We showed how cloud computing drives new WSN types, which introduces new directions, and discussed how WSN can coexist within social network domains. Compressed sensing was summarized next. We provided an overview of this important scheme and reviewed its utilization in WSNs. We proceeded with the MAC layer. Since the performance of many innovations in the higher layers rely on the underlay MAC protocol and since many data gathering schemes utilize traditional WSN MAC protocols, we provided an overview of these traditional protocols and mentioned state-of-the-art works in WSN and IoT. These works devise new MAC approaches for data gathering. Next, we covered the recent advances in routing. Similarly, we opened with an overview of the traditional routing protocols utilized for data-gathering and reviewed several recent enhancements. We reviewed the utilization of network coding for data gathering and explored the facilitation of UAV and mobile-sink in collecting the data from the sensors. Lastly, we turned our attention to the area of wearables which opens new research horizons for human health and activity surveillance and discussed the new paradigm of Wireless Body Area Networks. In the spirit of the times, we concluded with several studies that utilize some of the techniques discussed in this survey to aid in combating the COVID pandemic.

While we provided the general background to the research areas we covered, we mainly focused on cutting-edge research works. Yet we note that even seemingly exhausted topics, such as MAC and Routing protocols, provide new technological developments and present opportunities for new research domains.

As implied throughout this survey, there are several research areas that attract a lot of attention and anticipation for future developments. For example, technological innovations in manufacturing more compact sensing units with yet superior transmission, reception and processing capabilities are extremely in demand in several disciplines which include wearables, smart homes, IoT-related domains and others. All the more so, this is relevant when dealing with healthcare applications and implants. Such technological innovations will require in turn enhancements to other domains across the communication stack in order to adjust to the new opportunities and limitations. Energy acquisition is still a fruitful research domain. In this respect, finding new sources of energy harvesting (EH), better utilization of existing energy resources and energy storage are still challenging research fields. Similarly, new EH methods impose multiple new challenges on the entire protocol stack, which are correlated with the EH method, e.g., different EH methods dictate constraints on the MAC design, which in turn, impact the routing protocol which affects the performance end eventually the application utilizing the infrastructure. The growth of such networks supporting a variety of heterogeneous devices of communication standards and their increasing density requires more effective data compression techniques and efficient on-grid data analyses (i.e., even prior to data delivery to sinks). On the off-grid side of WSNs, we note that consistent progress in Cloud Computing (CC) technology and exploitation methodology will open new horizons in data analysis.

Deployment of Edge Computing units cooperating with sensing-capable units will imply the development of novel data-gathering schemes. As WSN density increases, challenges in the gathering of useful data by WSN and its consequent analysis will coincide with those of Big Data. Clearly, the processes of such analyses should be implemented within Cloud Computing systems. The fashion of the CC physical resources deployed in order to efficiently interact with WSN is not necessarily similar to those of usual IT-to-CC connectivity. It is not currently properly standardized and, most importantly, it is not clear how correctly CC (e.g., which HW, correct deployment of Access Points) should be cross-planned with a particular WSN. Issues of security and privacy which are not covered in this survey will continue to elicit a major interest, especially in keeping with evolving health care applications. The growth of the network and their variability will require a greater measure of adoption of ML and AI methods and the development of such new specialized methods for WSN in the very near future.
